# Oncolytic Viruses as In Situ Cancer Vaccines: Tumor Microenvironment Remodeling, Checkpoint Inhibitor Synergy, and Biomarker-Guided Clinical Translation

**DOI:** 10.3390/microorganisms14102385

**Published:** 2026-10-18

**Authors:** Kamil Rogalski, Michał Iwaszko, Anna Kuraś, Aleksandra Gałan, Mikołaj Dyrka, Przemysław Dybcio, Jan Ziemiński, Jakub Kleinrok, Agnieszka Korolczuk

**Affiliations:** 1 Department of Cardiac Surgery, Medical University of Gdansk, Marii Skłodowskiej-Curie 3a, 80-210 Gdansk, Poland; 2 Department of Clinical Pathomorphology, Medical University of Lublin, Jaczewskiego 8b, 20-090 Lublin, Poland; iwaszkomax@gmail.com (M.I.); aleksandra.galan00@gmail.com (A.G.); klejs.90@gmail.com (J.K.); 3 Faculty of Medicine, Medical University of Warsaw, Żwirki i Wigury 61, 02-091 Warsaw, Poland

**Keywords:** oncolytic viruses, cancer vaccines, tumor microenvironment, cell death, immune checkpoint inhibitors, biomarkers, precision medicine, genetic engineering

## Abstract

Oncolytic viruses (OVs) are increasingly viewed not only as tumor-lysing agents but also as immunotherapeutic platforms capable of functioning as in situ cancer vaccines. By selectively infecting malignant cells, inducing immunogenic cell death, releasing tumor-associated antigens and danger signals, and activating innate and adaptive immunity, OVs can remodel immune-desert or immune-excluded tumors. This narrative review integrates mechanistic and clinical evidence on tumor-selective viral replication, antigen presentation, dendritic-cell activation, type I interferon signaling, and the recruitment of cytotoxic T lymphocytes and natural killer cells. Particular attention is given to the effects of OVs on regulatory T cells, myeloid-derived suppressor cells, tumor-associated macrophages, cancer-associated fibroblasts, and extracellular matrix barriers. We further examine the biological rationale and clinical outcomes of combining OVs with immune checkpoint inhibitors, emphasizing the discrepancy between promising preclinical findings and inconsistent clinical efficacy. A stepwise translational framework is proposed to distinguish failures in delivery and replication from inadequate immune conversion, checkpoint-restrained immunity, and non-checkpoint-dominant resistance. Finally, we discuss antiviral immunity, safety, viral engineering, and tissue-based, circulating, and genomic biomarkers that may support patient selection and guide next-generation OV-based combination strategies.

## 1. Introduction

The acceleration in the development of cancer treatment methods observed over recent decades has contributed to growing interest in the use of viruses in this type of therapy. Viruses referred to as oncolytic viruses have been known for over a century. They are acellular infectious agents capable of lysing cancer cells, and considerable research hopes are associated with their therapeutic potential. The viruses discussed in this review preferentially replicate within malignant cells, leading to their destruction while relatively sparing healthy host cells. For the purposes of this article, we discuss oncolytic viruses as in situ cancer vaccines, defined as therapeutic agents that convert the patient’s own tumor into an endogenous source of antigens and immunostimulatory signals, thereby promoting systemic tumor-specific adaptive immunity. Antitumor activity of oncolytic viruses is based on two principal mechanisms: (1) direct destruction (lysis) of cancer cells and (2) induction of an immune response. Because of abnormalities in antiviral signaling pathways, particularly the interferon pathway, malignant cells are considerably more susceptible to viral infection. Following the lysis of an infected cell, the released viral particles infect other, previously uninfected cancer cells. At the same time, viral pathogen-associated molecular patterns (PAMPs) and cellular damage-associated molecular patterns (DAMPs), such as High-Mobility Group Box 1 (HMGB1) and ATP, are released, while calreticulin is exposed on the cell surface. These events may stimulate dendritic-cell activation, the release of pro-inflammatory cytokines, and increased activation of NK cells and CD8+ T lymphocytes. In the context of oncolytic virus therapy and their activity as in situ cancer vaccines, the successive stages in the development of an antitumor response can be presented in the following order: tumor-selective infection, immunogenic cell death, tumor-antigen release, antigen uptake and cross-presentation, tumor-specific T-cell priming, infiltration of non-injected lesions, epitope spreading and systemic immunity, and finally the development of immunological memory. Thus, evidence of oncolysis, DAMPs release, or innate immune activation alone should not be equated with the successful generation of an in situ cancer vaccine [1,2,3,4]. A notable example of an oncolytic virus is talimogene laherparepvec (T-VEC), a genetically modified HSV-1 virus that produces granulocyte-macrophage colony-stimulating factor (GM-CSF) and was approved by the Food and Drug Administration (FDA) in 2015 for the treatment of melanoma. The phase III OPTiM clinical trial (OncoVEX Pivotal Trial in Melanoma) demonstrated a markedly higher durable response rate than that observed in patients treated exclusively with GM-CSF. Modified adenoviruses are also currently being investigated in clinical trials. Another important example is ONYX-015, which has been evaluated in studies involving, among others, hepatocellular carcinoma and pancreatic cancer. In a 2021 mouse model of TC-1 tumors, Mozaffari Nejad et al. showed that intratumoral administration of an NDV-derived viral oncolysate inhibited tumor growth and enhanced antitumor immune responses, including cytotoxic T-cell activity and modulation of cytokine production [5]. Between 2000 and 2020, 1626 articles on oncolytic viruses were published; however, only a few oncolytic viruses have achieved commercial success and been approved as therapeutic products. A fundamental problem in assessing the efficacy of most oncolytic viruses remains the limited number of advanced clinical trials and the inconsistent translation of promising preclinical findings into clinical benefits [1,2,6,7,8]. In this article, we aim to summarize the available scientific evidence concerning oncolytic virus therapy combined with immune checkpoint inhibitors (ICIs), response biomarkers, the remodeling of immunosuppressive tumors toward an immune-responsive state, and the mechanisms of action of individual viruses, integrating these aspects into a single, coherent translational framework.

## 2. Methods

A targeted literature review was conducted using PubMed, Google Scholar, Web of Science, and Scopus to identify scientific publications relevant to the topic of this review. Articles published between January 2013 and August 2026 were considered, with an emphasis on ensuring that the majority of the included literature was recent and reflective of the current state of medical knowledge. The final literature search was conducted on 28 August 2026.

The primary aim of this review was to provide a comprehensive overview of oncolytic viruses, with particular emphasis on the biological basis of oncolytic viruses as in situ cancer vaccines, tumor microenvironment remodeling following oncolytic virotherapy, and the translational model of OV-induced immune conversion. The search concepts were mapped, where applicable, to the following MeSH descriptors: “Oncolytic Viruses,” “Cancer Vaccines,” “Tumor Microenvironment,” “Immunogenic Cell Death,” “Immune Checkpoint Inhibitors,” “Biomarkers,” “Precision Medicine,” and “Genetic Engineering.” The expressions “in situ cancer vaccination,” “antitumor immunity,” “biomarker-guided therapy,” and “engineered oncolytic viruses” were retained as free-text terms to preserve meanings not fully captured by individual MeSH descriptors. The initial literature search identified approximately 600 potentially relevant records using the aforementioned keywords and their combinations, prior to duplicate removal and subsequent screening.

The study selection process was performed in a structured but non-systematic manner, consistent with the narrative nature of the review. First, titles and abstracts were screened for relevance to oncolytic viruses and the biological mechanisms underlying tumor microenvironment remodeling or the use of oncolytic viruses as in situ cancer vaccines. Subsequently, the full texts of potentially eligible articles were assessed, with priority given to clinical trials, meta-analyses, observational studies, and guideline-based publications. Additional relevant articles were identified through manual screening of the reference lists of key publications. Our literature review was restricted to publications written in English or those for which an official English translation was available. The search focused primarily on the medical literature; however, relevant publications from the broader scientific literature were also considered when necessary to provide additional context and facilitate a more comprehensive understanding of the topics discussed.

Publications were included if they provided information on oncolytic viruses, their mechanisms of action, their potential applications in cancer therapy, or strategies for their genetic modification aimed at maximizing their therapeutic potential. Publications describing the molecular mechanisms underlying the tumor microenvironment and those involved in modulating the immunological sensitivity of tumor tissues were also included. Publications were excluded if they were considered outside the scope of the review, did not address the biological or therapeutic mechanisms of oncolytic viruses, or focused exclusively on topics beyond the thematic scope of this article. Duplicate publications were manually identified and removed during the literature screening and selection process. The literature search and study selection process is summarized in Table 1.

Because this was a narrative review, the evidence was synthesized qualitatively, and no formal systematic review protocol, PRISMA-based study selection procedure, or risk-of-bias assessment was performed.

## 3. Classification of Oncolytic Viruses

Oncolytic viruses may be classified as (1) DNA viruses or (2) RNA viruses. RNA and DNA viruses can be further divided into single-stranded (ss) and double-stranded (ds). RNA viruses are typically characterized by a smaller size (except for the remarkably small ssDNA parvovirus), higher genetic instability, and higher immunogenicity than DNA viruses [9]. Examples of ssRNA oncolytic viruses include measles virus, coxsackievirus group B, vesicular stomatitis virus, and Newcastle disease virus, while reovirus constitutes an example of a dsRNA virus. Common dsDNA virus candidates for oncolytic virotherapy (OVT) include adenovirus, HSV-1, and vaccinia virus [9,10].

They also can be divided into (1) viruses with natural lytic activity against tumors and (2) genetically modified viruses that acquire the previously described properties through genetic engineering. Viruses with natural tropism for cancer cells include measles virus, reovirus, vaccinia virus, coxsackievirus, echovirus, and poliovirus [1]. The genetic engineering of viruses aims not only to increase their tropism for cancer cells, but also to reduce toxicity to other cells and boost antitumor immune response [11]. Both DNA and RNA viruses can be modified; however, due to their larger transgene capacity and more stable genomes, DNA viruses are better candidates to be armed with genes encoding cytokines, ECM-degrading enzymes or other agents [12,13,14,15]. Examples of modified viruses include HSV-1, adenovirus, and vaccinia virus [1,16,17]. Genetically modified viruses are now being used increasingly often because they can be engineered to achieve the greatest possible antitumor activity [1,18]. Selected examples of these OVs are described in detail in Section 4.1.

## 4. Biological Basis of Oncolytic Viruses as In Situ Cancer Vaccines

### 4.1. Tumor-Selective Infection and Replication

The ability of oncolytic viruses to preferentially infect cancer cells is complex and depends on the specific type of virus. Some viruses that do not possess natural oncolytic properties must undergo appropriate genetic modifications to acquire the ability to selectively infect cancer cells. The most common mechanism by which these viruses enter target cells involves recognition of a specific membrane receptor expressed on the cell surface. As outlined in Section 8.1.1, surface receptor expression alone is sometimes insufficient to ensure viral permissiveness or therapeutic response, since tumor selectivity is multifactoral. Other factors, such as tumor lineage and transcriptional states, receptor accessibility (influenced by tumor microarchitecture and interstitial fluid pressure), also help regulate efficient infection [19,20,21].

One example is nectin-like protein 5 (CD155), a membrane receptor expressed at low levels on the surface of healthy cells but overexpressed in cancer cells. Overexpression of this protein is also associated with an unfavorable prognosis in cancer. In addition to naturally occurring proteins expressed by normally functioning cells, certain antigens are characteristic of cancer cells. These antigens may also serve as targets for oncolytic viruses engineered to bind selectively to them. Examples include EGFR and CD20 [22]. Another mechanism underlying the preferential targeting of cancer cells over healthy cells is the selective replication of these viruses in malignant cells. Owing to their natural properties or introduced genetic modifications, these viruses can overcome cellular responses that restrict replication while remaining unable to inhibit the antireplicative mechanisms of healthy cells. Herpes simplex virus (HSV) provides an example of this mechanism. Following deletion of the neurovirulence gene ICP34.5, which inhibits the host protein kinase R (PKR) pathway, the virus exhibits a limited capacity to replicate in healthy cells. At the same time, its replication is enhanced in cells with alterations in the PKR pathway [18,22].

Abnormalities in the interferon response of cancer cells constitute a particularly important aspect of oncolytic virus activity. Defects in this component of the antiviral response may impair the establishment of an effective antiviral state and consequently facilitate viral replication [22,23]. Study conducted by Ch’ng et al. in 2013 [24], the AF2240 strain of Newcastle disease virus is capable of modulating the interferon response in cancer cells. Selective apoptosis of these cells was associated with the production of IFN-β in the absence of IFN-α production. Activation of the p38 MAPK/NF-κB/IκBα pathway is believed to account for this response [23,24]. The importance of pre-existing abnormalities in the interferon response for oncolytic virus replication was also demonstrated by a 2000 study of vesicular stomatitis virus (VSV) [25]. An intact interferon response limited viral production in normal cells, whereas cancer cells with defects in this pathway remained permissive to viral replication [25]. Another study, published in 2020, demonstrated that homozygous deletion of type I interferon genes enhanced the lytic activity of measles virus against mesothelioma cells. At the same time, some cancer cells retained the ability to recognize the virus and respond to infection through the nuclear translocation of interferon regulatory factor 3and NF-κB. This enabled the induction of a partial interferon response that reduced their susceptibility to the virus [22,26]. Taken together, these examples indicate that both impaired interferon responses in cancer cells and the ability of certain viruses to modulate host antiviral mechanisms may promote viral replication and enhance direct oncolytic activity.

Several selected examples of these platforms are presented below.

#### 4.1.1. Adenovirus

Adenoviruses are double-stranded DNA viruses with a genome of approximately 35 kb. Their relatively large genome enables the incorporation of long DNA sequences and therefore permits extensive genetic modification. Adenoviruses can be divided into 57 distinct serotypes classified into species A through G. The most commonly used platforms are the Ad3 and Ad5 subtypes. Most of modern oncolytic adenoviruses are based on Ad5 subtype. These viruses enter cells primarily through the coxsackievirus and adenovirus receptor [1,2,27]. Coxsackievirus and adenovirus receptor-mediated attachment initially facilitates the interaction of cell-surface integrins with arginine–glycine–aspartic acid motif motifs located in penton proteins at the capsid vertices. However, cancer cells exhibit reduced expression of this receptor on their surface, which represents a therapeutic challenge. Adenoviral transcription in the host cell requires two key viral genes: (1) E1A and (2) E1B. The former activates transcription of the E2F factor and stimulates the transition of the host cell from a quiescent state into the cell cycle. The E1B gene encodes the E1B-55 kDa protein, which promotes p53 degradation, and the E1B-19 kDa protein, which inhibits premature apoptosis of virus-infected cells [1]. High population seropositivity against adenoviruses also appears to be an important limitation of their therapeutic use. This is directly related to their previously mentioned widespread occurrence in humans and animals. The first oncolytic virus approved for cancer therapy was developed from an adenovirus. This virus was H101 (Oncorine), approved in 2005 by the Chinese State Food and Drug Administration as a therapeutic option for head and neck cancer. It contains a deletion of the gene encoding the previously described E1B-55 kDa protein in adenovirus type 5 (Ad5). The virus was designed to selectively replicate in cancer cells with abnormalities in the p53 pathway, although its tumor selectivity is now considered to involve additional mechanisms [1,28]. Numerous studies are currently investigating new therapeutic applications of adenoviruses. These include studies of their use in glioblastoma and high-grade glioma. DNX-2401 has undergone early-phase clinical evaluation, and studies conducted to date have yielded promising findings regarding its use in anticancer therapy, as supported by a 2018 study published in JCO, in which 20% of patients survived for more than three years following intratumoral injection [28,29,30]. Adenoviruses are also being considered as therapeutic options for, among others, hepatocellular carcinoma, pancreatic cancer, bladder cancer, prostate cancer, and biliary tract cancer [1,2,3,26,29,30].

#### 4.1.2. Herpes Simplex Virus

To date, HSV-1 has been the most successful platform among oncolytic viruses. It is a double-stranded DNA alphaherpesvirus with a large genome of 152 kb that naturally infects sensory cells and resides in sensory ganglia. The T-VEC platform mentioned in the introduction is currently the only oncolytic virus approved for therapeutic use by both the FDA, in 2015, and the European Medicines Agency, and it has been used in the treatment of advanced stage III and IV melanoma. The most important modification of this virus is the previously described deletion of the neurovirulence gene ICP34.5, which inhibits the cellular PKR pathway. Other modified genes include: (1) ICP47—its deletion increases MHC class I presentation on cells by preventing ICP47-mediated inhibition of the transporter associated with antigen processing, thereby enhancing the presentation of cancer cells to CD8+ T lymphocytes; and (2) GM-CSF—insertion of a human copy of this gene in place of the ICP34.5 gene stimulates local cytokine production within the tumor microenvironment, thereby promoting a local immune response. HSV-1 particles are thought to enter cells through nectin-1, nectin-2, and herpesvirus entry mediator [1,31]. Studies are currently evaluating combination therapy with T-VEC and immune checkpoint inhibitors (ICIs), such as ipilimumab and pembrolizumab. It is worth noting that a double-blind study published in 2021 did not demonstrate a particular benefit of combining T-VEC with pembrolizumab compared with placebo [17,18]. Another oncolytic virus in this group is G47∆, which carries three mutations involving the deletion of ICP34.5, ICP6, and α47 and is the first virus of this type approved for the treatment of brain tumors. In an in vivo model involving mice implanted with glioma cells, it effectively reduced the rate of tumor growth and improved survival in the study group. A phase I/II clinical trial involving 13 patients with glioblastoma was conducted between 2009 and 2014 at the University of Tokyo Hospital and the hospital of the Institute of Medical Science, University of Tokyo, and yielded promising results. Following two injections of the preparation, the one-year survival rate was 38.5%. The findings of this study provided the basis for further research on G47∆ [17,30,31]. Between 2015 and 2018, a single-arm phase II clinical trial was conducted in 19 patients, who received up to six injections of the preparation. The one-year survival rate in this cohort was 84.2% [32].

#### 4.1.3. Vaccinia Virus

Vaccinia virus is also classified as a DNA virus and belongs to the family Poxviridae. It has a large, double-stranded DNA genome. It exhibits strong tropism for cancer cells. An unquestionable advantage of vaccinia virus is its ability to replicate exclusively in the cytoplasm of infected cells, thereby eliminating the potential risk of insertional mutagenesis [1]. Another advantage of vaccinia virus is its rapid lytic replication cycle. Virions are released from infected cells as early as eight hours after infection, whereas cell lysis occurs after approximately 48–72 h. Viral replication can also occur under hypoxic conditions [33]. JX-594 is a vaccinia virus platform that is clearly worthy of mention. It contains a deletion of the thymidine kinase gene. Considerable hopes were placed on JX-594 in the treatment of hepatocellular carcinoma. However, the phase III PHOCUS trial, which was designed to compare the efficacy of JX-594 combined with sorafenib against conventional sorafenib monotherapy, was terminated because of a lack of efficacy [31].

#### 4.1.4. Reovirus

RNA viruses may also serve as potential oncolytic platforms. Reovirus is a non-enveloped, double-stranded RNA virus. A finding that supported consideration of reovirus as a potential anticancer therapy was the demonstration of its tropism for cells expressing the epidermal growth factor receptor (EGFR) and that activation of downstream signaling pathways increased the likelihood of cell infection. However, the mechanism underlying the preferential infection of cancer cells by this virus appears to be multifactorial and is not yet fully understood. Reovirus can induce apoptosis through the S1 and M2 genes, which encode the outer capsid proteins σ1 and μ1. These proteins activate tumor necrosis factor (TNF), tumor necrosis factor-related apoptosis-inducing ligand (TRAIL), and Fas-mediated death receptor signaling [1,18,34]. Reovirus is currently regarded as a promising treatment for hematological malignancies, such as multiple myeloma, Burkitt lymphoma, and diffuse large B-cell lymphoma [34]. Another research direction worthy of mention concerns the efficacy of reovirus in combination therapy for stage IV gastric cancer. Mori et al. demonstrated that combining reovirus therapy with irinotecan, paclitaxel, or docetaxel enhanced cytotoxic activity in vitro. In a mouse model, reovirus monotherapy limited tumor growth, whereas combination therapy further enhanced the antitumor effect [35].

#### 4.1.5. Measles Virus (MV)

It is a negative-sense RNA virus belonging to the Paramyxoviridae family. Vaccine strains have become well established in medicine worldwide and have enabled highly effective prevention of measles. These strains, specifically the Edmonston B lineage, were used to construct an oncolytic measles virus by inserting the human sodium iodide symporter (NIS) gene [36]. This gene enables non-invasive monitoring of viral activity during therapy. One of the receptors used by the virus to enter host cells is CD46. This receptor is overexpressed on the surface of cancer cells, thereby increasing the tropism of the oncolytic virus. CD46 expression on the surface of healthy cells is lower, resulting in a reduced tendency of the virus to infect them. Cancer cell lysis occurs through virus-induced cell-to-cell fusion. This process leads to the formation of multinucleated syncytia, which subsequently die [1,2,31,36].

Measles virus is also being investigated as a potential therapeutic option for biliary tract cancer through the insertion of the super cytosine deaminase (SCD) gene. SCD is a fusion protein comprising yeast cytosine deaminase and uracil phosphoribosyltransferase. It converts 5-fluorocytosine into 5-fluorouracil and ultimately into 5-fluorouridine monophosphate. Under in vitro conditions, MV-SCD combined with 5-fluorocytosin enhanced the cytotoxic effect against biliary tract cancer. In two in vivo mouse xenograft models, treatment with this virus reduced tumor size in the first model and also improved survival in the second [2,37].

The OV platforms discussed in detail in this review were selected as representative rather than exhaustive examples. Numerous additional viral platforms have been investigated for oncolytic potential, including Newcastle disease virus, vesicular stomatitis virus, coxsackievirus A21, H-1 parvovirus, poliovirus-based vectors, and Seneca Valley virus. A detailed discussion of each of these platforms would require a substantially broader, platform-focused review.

### 4.2. Immunogenic Tumor Cell Death and Antigen Release

The main desired effect of oncolytic viruses is the lysis of cancer cells, followed by the destruction of the tumor mass, potentially leading to remission. A major challenge to their oncolytic activity is the immunosuppressive nature of the tumor microenvironment. This immunosuppression is mediated by multiple factors, including high numbers of regulatory T cells and elevated levels of inhibitory cytokines such as IL-10 and TGF-β. Abnormal vascular remodeling and a denser extracellular matrix (ECM) also impede the lytic activity of the viruses discussed in this review [38]. The effects of oncolytic viruses on these components of the immunosuppressive tumor microenvironment will be discussed in subsequent sections of this article.

Oncolytic viruses induce cancer cell death through: apoptosis, autophagic cell death, pyroptosis, necroptosis, ferroptosis and PANoptosis. Some of these processes take the form of immunogenic cell death (ICD) [3,17,31,39,40].

ICD is a form of regulated cell death capable of inducing an adaptive immune response directed against antigens derived from dying cells. In the context of oncolytic virus infection, this process is accompanied by the release of tumor-associated antigens, the exposure or release of damage-associated molecular patterns (DAMPs) derived from cancer cells, and the presence of viral pathogen-associated molecular patterns (PAMPs). The DAMPs emitted by infected cells depend on: (1) the type of virus with which the cells are infected and (2) the type of tumor.

The principal DAMPs emitted by cancer cells include: (1) calreticulin (CRT), (2) ATP, and (3) high-mobility group box 1 (HMGB1) [3,17,31,33]. Each of these molecules has a specific temporal window during which it actively contributes to tumor oncolysis.

#### 4.2.1. Calreticulin (CRT)

Calreticulin is a chaperone protein involved in the regulation of cellular calcium homeostasis. It is exposed on the cell surface between 6 and 12 h after infection. CRT translocates toward the plasma membrane by forming complexes with the protein disulfide isomerase ERp57. The receptor expressed on immune cells that binds CRT exposed on the cell surface has not yet been fully identified. Candidate receptors include CD91, scavenger receptor expressed by endothelial cells I, and scavenger receptor class A, which are expressed on dendritic cells and other cells involved in innate immune responses. CRT functions as an “eat me” signal for the immune system. This signal is counteracted by CD47, which provides a “don’t eat me” signal [31,41,42,43].

#### 4.2.2. ATP

ATP release from infected cells begins between 12 and 24 h after infection. This process involves activated pannexin-1 channels and increased plasma membrane permeability resulting from viral activity. Pannexin-1 channels can be opened, among other mechanisms, through activation of the caspase-3/-7 pathway [31,41,42,44]. Secreted ATP also contributes to the recruitment of immune cells into the antitumor response. This effect is mediated by the purinergic receptors P2Y2 and P2X7, which are activated by extracellular ATP [31]. ATP also induces assembly of the NLRP3 inflammasome, thereby stimulating the secretion of IL-1β and IL-18. IL-1β, in turn, enhances the secretion of chemokines such as CXCL1 and CCL2, further promoting the activation of immune cells [31,41,43].

#### 4.2.3. HMGB1

HMGB1 is released from dead cancer cells approximately 24–48 h after infection with an oncolytic virus. This protein can bind DNA molecules within the cell nucleus. However, following its release into the extracellular space during cell death, HMGB1 binds to Toll-like receptor 4 (TLR4) and the receptor for advanced glycation end products expressed on dendritic cells [31,45]. Following the recognition of HMGB1 by TLR4, the transcription of the gene encoding the adaptor molecule myeloid differentiation primary response 88 (MyD88) is induced. Ultimately, activation of the TLR4–MyD88 cascade inhibits lysosome–phagosome fusion, thereby affecting the processing of phagocytic vesicles in dendritic cells and accelerating the uptake of tumor antigens by these cells [42].

### 4.3. Innate and Adaptive Antitumor Immunity

One of the key consequences of ICD is the activation of dendritic cells, which represents an important step linking innate and adaptive antitumor immunity. The production of type I interferons during viral infection, including by infected tumor cells, plays an important role in this process. This process occurs approximately 6–24 h after infection with an oncolytic virus [31]. The effects of type I interferons on NK cells and CD8+ T lymphocytes will be discussed in greater detail in the following chapter.

## 5. Tumor Microenvironment Remodeling After Oncolytic Virotherapy

### 5.1. Conversion of Cold Tumors into Inflamed Tumors

Characteristics of cold tumors include an immunosuppressive microenvironment and the absence or minimal infiltration of CD8+ T lymphocytes. These tumors can be divided into various immunological phenotypes. In immune-excluded tumors, T-cells are present at the periphery but because of physical barriers fail to infiltrate the tumor mass. Their infiltration and survival within the tumor mass are blocked due to stroma produced by cancer-associated fibroblasts (CAFs), along with abnormal angiogenesis and localized hypoxia. The immune-dessert phenotype is defined by a complete lack of immune cells in the whole tumor. Cold tumor microenvironment can also present as an immunosuppressive subtype, characterized by the presence of suppressive cells, such as tumor-associated macrophages (TAMs), myeloid-derived suppressor cells (MDSCs), and regulatory T cells (Tregs), as well as inhibitory cytokines, including IL-10 and TGF-β. Clinically, these factors are associated with a poor prognosis and a less favorable response to immune checkpoint inhibitors [31,46].

To overcome these barriers, oncolytic virotherapy has been adopted as a strategy that can be employed to convert immune-excluded and immune-desert tumors into immune-inflamed phenotypes. Immunogenic cell death causes the release of DAMPs. Concurrently, the viral infection also leads to the release of PAMPs in the tumor microenvironment (TME), as well as increased production of chemokines, such as CXCL9 and CXCL10, which stimulate the migration of effector T-cells [31,47]. This results in the maturation of dendritic cells (DCs) and increased T cell and NK cell infiltration into the TME [48,49]. Additionally, oncolytic viruses can also reduce local immunosuppression through modulation of Tregs, MDSCs, and TAMs. Furthermore, they contribute to the breakdown of stromal barriers through targeting CAFs and extracellular matrix (ECM) degradation [50,51,52,53]. OV-mediated induction of inflammation and immune cell infiltration enhance tumor susceptibility to immune checkpoint therapy [46]. Changes in the TME induced by oncolytic viruses are summarized in Table 2.

### 5.2. Effects of Oncolytic Virotherapy on Effector Immune Cells

#### 5.2.1. Effects on CD8+ T-Cells

Following the maturation of DCs, these cells migrate to the draining lymph nodes, where cross-presentation of antigens and priming of CD8+ T-cells occurs. Additionally, DCs secrete type I IFNs, pro-inflammatory cytokines (including IL-1β, IL-6, IL-12 and TNF-α), and chemokines (IL-8, CCL2, CXCL10) as a result of the activation of pattern recognition receptors due to PAMPs. This process establishes chemotactic gradients that result in the recruitment of CD8+ T cells into the TME [48,54,55]. The previously mentioned local release of type I IFNs leads to the activation of CD4+ cells. Further, CD4+ Th1 cells produce TNF and IFN-γ, increasing the cytotoxic effect of tumor-specific CD8+ T cells. Moreover, type I IFNs directly upregulate the expression of MHC class I on tumor cells, making them sensitive to CD8+ T cell recognition [31,48]. Ultimately, OVT can lead to deep and stable infiltration of cytotoxic T cells. This antitumor immune response is sustained through the formation of memory-like T cells located in the lymphoid clusters [13]. In order to intensify these immunologic changes, OVs can be engineered to express specific cytokines that further enhance T-cell migration into the TME [56]. Hatimi et al. demonstrated in mouse models that arming an OV with IL-12 promotes the expansion of a CD8+ T cell subpopulation with a long-lived effector cell phenotype, resulting in superior therapeutic antitumor efficacy compared to the unarmed virus [14].

#### 5.2.2. Effects on NK Cells

The role of NK cells in OVT remains ambiguous. On the one hand, NK cell activation results in the direct lysis of tumor cells. On the other hand, their potent antiviral response may negatively affect the outcome of treatment by causing early elimination of the virus [57]. Infection of tumor cells by OVs leads to enhanced cytotoxicity of NK cells. Leung et al. showed that interaction with infected cells activates DNAX accessory molecule-1 (DNAM-1) on NK cells, thus accelerating the elimination of infected tumor cells and increasing cytotoxicity of NK cells against uninfected malignant cells [58]. Additionally, type I IFN signaling pathway causes NK cells to enter into the antiviral state, protecting them from direct infection and stimulating their antitumor potential against both infected and uninfected cells. This is achieved through the upregulation of TRAIL on NK cells, which binds to TRAIL-R1 and TRAIL-R2 on tumor cells, leading to their apoptosis [59]. Besides the death receptor-mediated pathways, NK cells eliminate tumors by releasing perforin and granzymes. To maximize this effect, therapeutic approaches employ OVs engineered to express immunosuppressive antagonists. The inclusion of TGF-β inhibitor has been shown to significantly increase the production of granzyme B and perforin, enhancing the overall antitumor activity of NK cells [49,60]. Furthermore, type I IFNs reduce IFN-γ secretion, serving as an important regulatory mechanism. This suppression is beneficial because excess IFN-γ could otherwise stimulate the expression of NK cell inhibitory ligands on tumor cells, increasing their resistance to NK-mediated killing [59].

### 5.3. Effects of Oncolytic Virotherapy on Suppressive Immune Cells and Stromal Barriers

#### 5.3.1. Effects on Tregs

Regulatory T cells constitute an important immunosuppressive population that enhances immune escape through the secretion of IL-10 and TGF-β. These cytokines inhibit the activity of effector T cells and NK cells [61]. The OVT represents a tool to fight this suppression by recruiting intratumoral CD8+ T cells without expanding the Treg population, thereby improving the CD8/Treg ratio [62]. Although unmodified OVs may not directly deplete the population of Tregs, they can be used for delivering agents that locally reduce immunosuppression, for instance, TGF-β inhibitors. The localized delivery significantly reduces the risks associated with toxicity caused by the systemic administration [50,62]. Studies conducted by DePeaux et al. revealed that OV-induced IFN-γ can drive Tregs into a fragile phenotype, limiting their suppressive capacity. However, heavily immunosuppressive tumors usually exhibit high concentrations of TGF-β, which results in more stable Tregs, resistant to IFN-γ. The use of an oncolytic virus armed with TGF-β inhibitor allows to overcome this resistance, thereby promoting Treg fragility and resulting in an environment more responsive to immunomodulation [50].

#### 5.3.2. Effects on MDSCs

Myeloid-derived suppressor cells are well-established mediators of tumor immune escape, largely through the expression of programmed death-ligand 1 (PD-L1), secretion of inhibitory cytokines and promotion of Treg expansion. In addition, the polymorphonuclear MDSCs (PMN-MDSCs), primarily located in the peripheral lymphoid organs, induce immunosuppression by generating reactive oxygen species. This subset has been identified to be a major barrier to the efficacy of OVT [51,61]. Oncolytic viruses stimulate the release of C-X-C chemokines within the TME. This immune reaction attracts C-X-C motif chemokine receptor 2 (CXCR2)-expressing PMN-MDSCs to the tumor site. When they localize in the tumor area, these cells suppress NK cell activity and secrete IL-10, which impairs dendritic cell function and inhibits effector T cell activation. Combination of OVT with agents that prevent PMN-MDSC recruitment represents a promising strategy to enhance therapeutic efficacy [51].

#### 5.3.3. Effects on TAMs

TAMs consist of various subsets that constitute a spectrum of proinflammatory, mixed, or protumoral phenotypes. They exhibit substantial functional, transcriptional, and spatial heterogeneity. For instance, osteopontin-expressing (SPP1+) TAMs are more abundant in hypoxic and necrotic areas of the tumor. This subset exhibits immunosuppressive properties, stimulates angiogenesis, and promotes matrix remodeling. Another subpopulation that enhances immunosuppression is the CCL18+ TAM subset, which increases Treg recruitment. Additionally, triggering receptor expressed on myeloid cells 2 (TREM2+) TAMs suppress CD8+ T cell infiltration through the downregulation of CXCL9. In contrast, ficolin-1-positive (FCN1+) TAMs participate in antigen presentation and exhibit proinflammatory activity [63]. The administration of OVs causes activation of the antiviral immune response mediated by systemic macrophages. Through active phagocytosis and the secretion of type I IFNs, these cells can limit the spread of OVs, thereby decreasing therapeutic efficacy. Additionally, the viral infection upregulates CCL2 expression, which increases TAM infiltration into the TME [22]. Furthermore, OVT induces proinflammatory functions in TAMs. The viral infection promotes the release of extracellular vesicles containing PAMPs and DAMPs. This increases the secretion of TNF-α and IL-1, as well as the expression of costimulatory molecules CD80 and CD86 on TAMs, thereby shifting them toward proinflammatory phenotypes and amplifying the local inflammatory response [32,64].

#### 5.3.4. Effects on CAFs and Extracellular Matrix

CAFs and ECM are non-immune stromal components that induce immunosuppression and promote therapeutic resistance. CAFs are associated with matrix production and remodeling, as well as the secretion of immunosuppressive factors, such as TGF-β. The ECM is mostly composed of collagen, fibronectin, and proteoglycans, and functions as a physical barrier that limits the penetration of immune effector cells, antineoplastic drugs, and oncolytic viruses into the TME [61,65]. The effect of OVT on these stromal elements can be mediated by either direct viral cytotoxicity or specific compounds delivered by modified viruses. For instance, Kurisu et al. demonstrated that oncolytic reovirus directly induces CAF apoptosis in the TME of mice [66]. The use of viruses engineered to express matrix-degrading proteins, such as hyaluronidase, decorin or relaxin are able to dismantle the ECM. This structural degradation not only amplifies the antitumor efficacy of OVT but also allows the recruitment of immune cells and improves the intratumoral distribution of chemotherapeutics and immune checkpoint inhibitors [15,65]. Furthermore, since CAFs characteristically overexpress fibroblast activation protein-α (FAP), this molecule serves as a potential target for intervention. Thus, oncolytic viruses armed with FAP-targeted bispecific T-cell engager induce the activation and proliferation of T cells, resulting in the destruction of CAFs by these immune cells. Ultimately, this approach leads to stromal degradation and boosts antitumor therapeutic efficacy [53,67].

**Table 2:** Main TME changes induced by oncolytic viruses.

Tumor Microenvironment Component	Main Effects Induced by OVs
CD8+ T cells	Recruitment and increased infiltration into the TME, amplified cytotoxicity, enhanced recognition of tumor cells through MHC class I upregulation, generation of memory-like T cells [13,31,48,54,55]
NK cells	Amplified cytotoxicity through DNAM-1 and upregulation of TRAIL, increased production of perforin and granzymes [13,31,48,54,55]
Tregs	Improved CD8+/Treg ratio, transition into a fragile phenotype with limited suppressive capacity [50,62]
MDSCs	Context-dependent effects: OV-induced C-X-C chemokines can recruit suppressive PMN-MDSCs into the TME, whereas oncolytic reovirus can reduce MDSC-mediated suppression of CD8+ T-cell proliferation through a TLR3-dependent mechanism [51,68,69].
TAMs	CCL2-associated recruitment and potential shift in polarisation from protumoral towards more proinflammatory phenotypes; however, macrophage-mediated phagocytosis and type I IFN production can restrict viral persistence and spread, limiting therapeutic efficacy [52,70,71].
CAFs and ECM	Induction of CAF apoptosis, degradation of the extracellular matrix [39,53,65,66]

Abbreviations: TME: tumor microenvironment; OVs: oncolytic viruses; NK cells: Natural Killer cells; DNAM-1: DNAX accessory molecule-1; TRAIL: TNF-related apoptosis-inducing ligands; Tregs: regulatory T cells; MDSCs: myeloid-derived suppressor cells; PMN-MDSCs: polymorphonuclear myeloid-derived suppressor cells; TAMs: tumor-associated macrophages; CCL2: C-C motif chemokine ligand 2; CAFs: cancer-associated fibroblasts; ECM: extracellular matrix.

### 5.4. Morphological Changes in Tumor and Peritumoral Tissues

Histopathological changes following oncolytic virotherapy reflect both tumor destruction and inflammatory remodeling, with findings varying according to the viral platform, tumor type, and timing of tissue sampling. Within the tumor, documented manifestations include necrosis, loss of viable malignant cells, and virus-associated cytopathic changes. In recurrent malignant glioma treated with DNX-2401 [16], resected specimens demonstrated a necrotic core bordered by an area of active adenoviral replication and a peripheral compartment of tumor cells not yet infected. Viral protein expression was accompanied by prominent inclusion bodies, providing morphological evidence of productive infection [16].

The tissue response also involves the surrounding stroma and adjacent non-neoplastic structures. In T-VEC-treated melanoma, biopsies have shown T-cell and plasma-cell infiltrates, histiocytic reactions, and granuloma formation. Inflammation may exhibit perivascular, interstitial, or perineural distributions, extending beyond residual tumor nests. These reactive changes do not necessarily indicate ongoing viral infection of surrounding normal tissue: in one histopathological series, HSV-1 immunohistochemistry was negative in the reactive infiltrates and surrounding tissue examined [72].

Regressing melanoma lesions may additionally show fibrosis and aggregates of pigment-laden macrophages, termed tumoral melanosis when viable melanoma cells are absent. Such changes can persist despite tumor clearance and may clinically resemble residual disease. Histological examination, supplemented by appropriate tumor-specific immunohistochemistry, can therefore help distinguish treatment-related inflammation and regression from persistent malignancy [73]. These observations illustrate why morphological findings should be interpreted in the context of the specific treatment and sampling site.

## 6. Translational Model of OV-Induced Immune Conversion

### 6.1. When OV Therapy May Act as an Effective In Situ Vaccine

**Figure 1:**
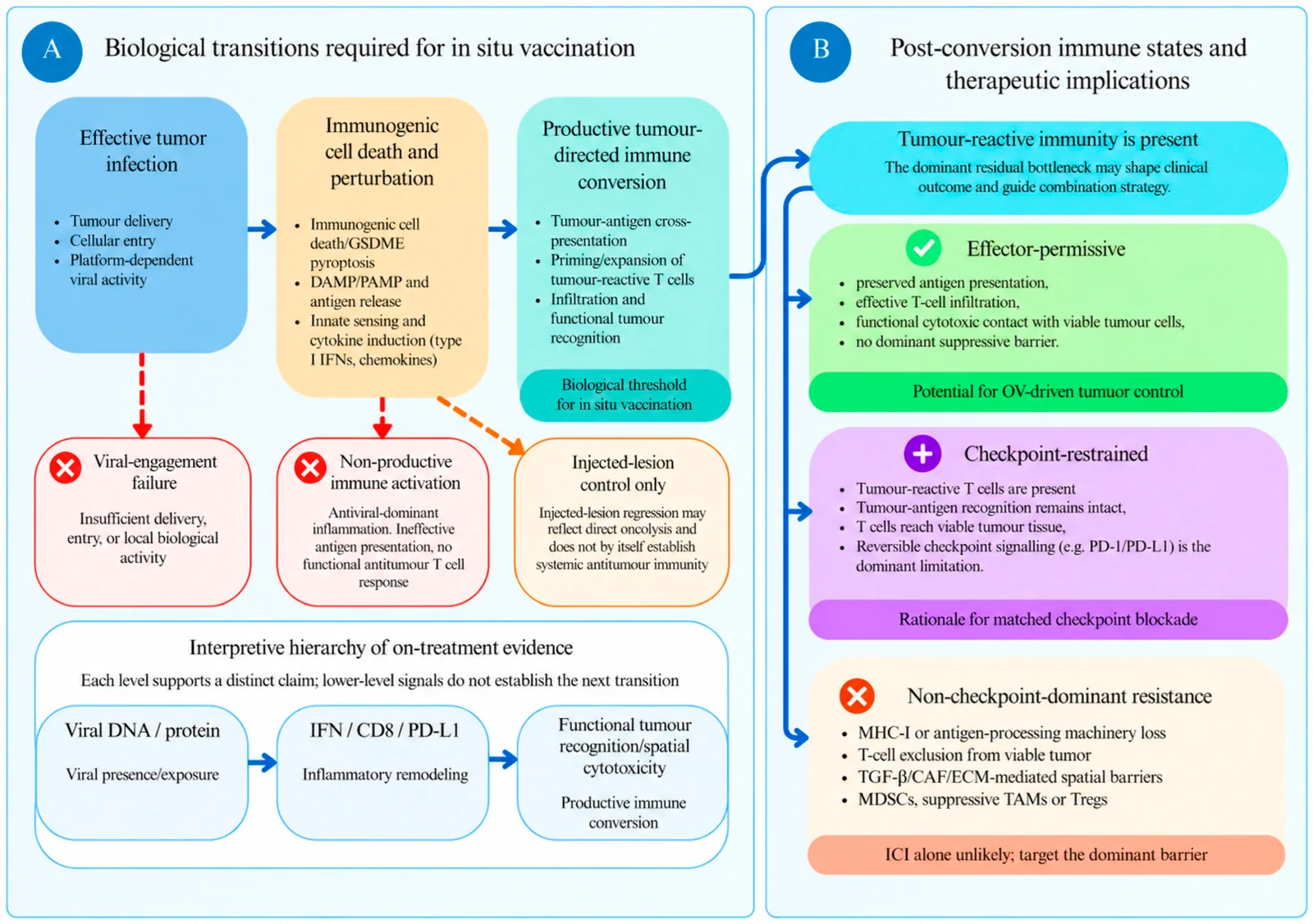
Translational model of oncolytic virus-induced immune conversion. (**A**) Biological transitions, potential points of failure, and the interpretive hierarchy of on-treatment biomarkers. (**B**) Post-conversion immune phenotypes and their therapeutic implications.

Successful OV-based therapy requires three consecutive biological transitions: effective tumor infection, immunogenic cell death and perturbation, and productive tumor-directed immune conversion (Figure 1A). Each step has distinct determinants and represents a potential point of failure. When productive immune conversion succeeds, the TME may assume one of three proposed functional states: effector-permissive, checkpoint-restrained or non-checkpoint-dominant resistance (Figure 1B). These states may inform about the dominant residual bottlenecks and guide therapeutic implications, dictating whether an OV alone is sufficient or if a rational combination strategy with immune checkpoint inhibitors (ICIs) is required.

#### 6.1.1. Effective Tumor Infection

**Figure 2:**
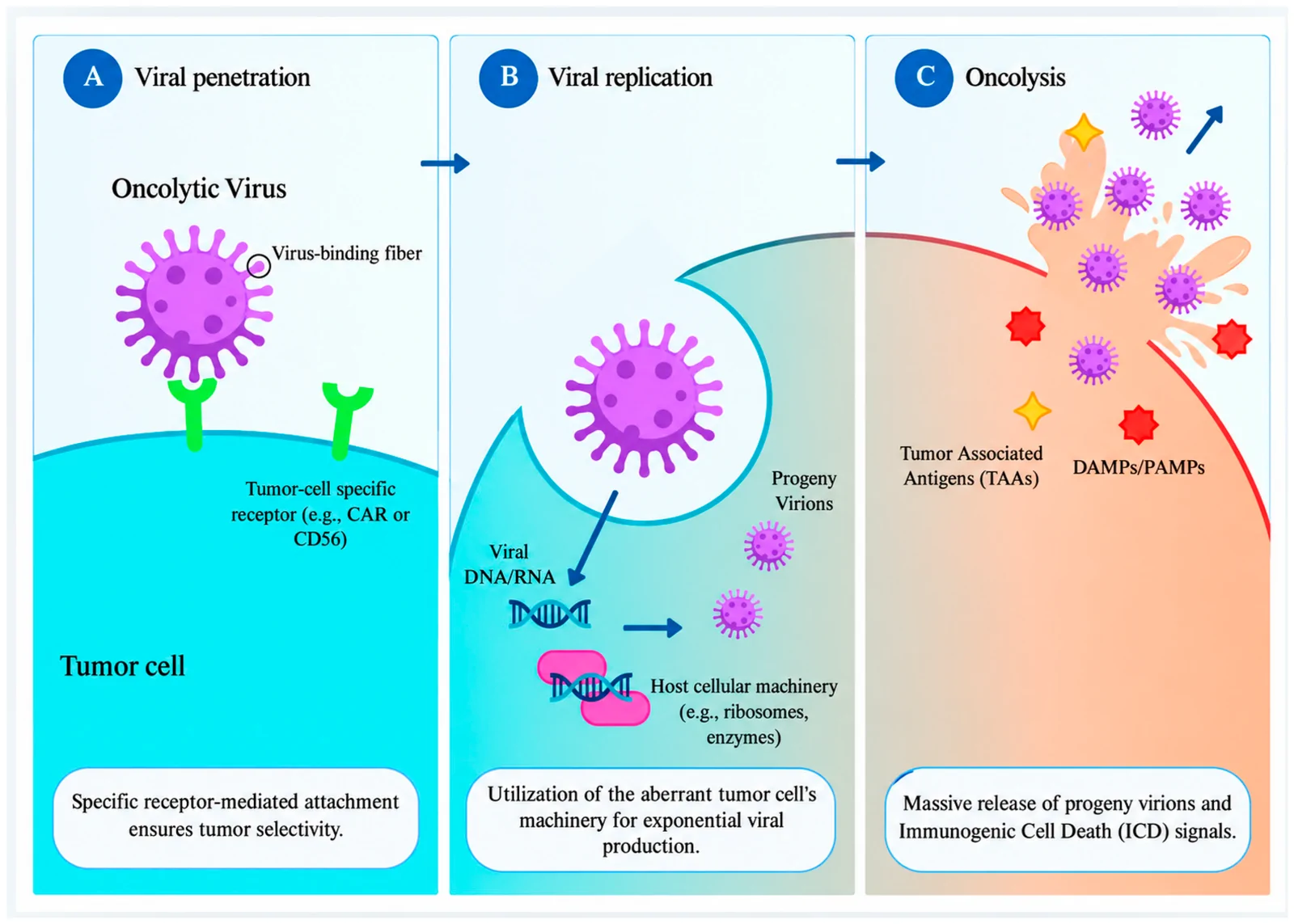
Basic mechanism of oncolytic virus (OV)-induced tumor cell lysis. (**A**) Viral penetration: The oncolytic virus, under favorable microenvironmental conditions binds to specific receptors (e.g., CAR or CD46) overexpressed on the tumor cell surface, ensuring tumor selectivity prior to internalization. (**B**) Viral replication: Following entry, the virus releases its viral DNA/RNA and hijacks the host cellular machinery (such as ribosomes and enzymes) to synthesize viral proteins and replicate its genome, driving the exponential production of progeny virions. (**C**) Oncolysis: The accumulation of viral particles causes lytic cell rupture. This burst releases not only new progeny virions capable of infecting neighboring cancer cells, but also massive immunogenic cell death (ICD) signals—specifically damage-associated molecular patterns (DAMPs), pathogen-associated molecular patterns (PAMPs), and tumor-associated antigens (TAAs)—which are crucial for initiating the subsequent antitumor immune response.

The process of selective tumor cell lysis is initiated by binding of the virus to specific receptors overexpressed on cancer cell surfaces (Figure 2A). Following entry, OVs hijack the host cellular organelles to synthesize viral proteins and replicate their genome (Figure 2B). This process results in the accumulation of viral particles, subsequent cell rupture, and release of progeny virions capable of infecting additional cancer cells (Figure 2C) [74].

Productive infection more broadly requires convergence of several tumor-intrinsic factors beyond receptor expression: high metabolic/replicative activity that favors viral replication machinery; oncogenic driver mutations, such as activated Ras or Akt signaling exploited by reovirus and myxoma virus, respectively; and tumor suppressor loss, for example that of p53, ATM, Rb, that increases permissiveness and defective type I interferon signaling, which is characteristic of many malignant cells and allows selective viral replication relative to interferon-competent normal tissue [75,76,77]. For some virus families, tumor cell expression of specific processing enzymes required for virion maturation is an additional prerequisite [78].

Not achieving these conditions leads to viral-engagement failure, characterized by insufficient delivery, entry, or local biological activity. In tumors a constitutive interferon pathway activation is itself a determinant of resistance, while simultaneously fostering selective viral replication in malignant cells, thereby representing potential predictive biomarkers of OV susceptibility, since interferon signaling is a double-sided determinant. In glioblastoma patient-derived xenografts this signature predicted resistance to oncolytic measles virus independent of receptor expression, and pharmacologic JAK inhibition could reverse resistance and increase viral yield 1000-fold in vitro [79]. Similarly, low constitutive STAT1/interferon-stimulated gene expression in melanoma sensitized tumors to a novel oncolytic virus and correlated with subsequent oncolytic potency [80]. Moreover, interferon pathway status and defects in innate viral sensing are increasingly regarded as candidate predictive biomarkers of OV sensitivity across tumor types, not solely in glioblastoma or melanoma models [81,82]. In the context of on-treatment monitoring, the detection of viral DNA or protein reflects viral presence and exposure, representing the initial baseline level of evidence rather than functional immune conversion.

#### 6.1.2. Productive Immune Conversion

Successful infection alone is insufficient to achieve systemic efficacy. Oncolysis must trigger immunogenic cell death (ICD) and perturbation, which serves as the prerequisite to achieve productive tumor-directed immune conversion. Several OVs generate antigen-specific T-cell immunity through this route, including HSV-1, vesicular stomatitis virus (VSV), and oncolytic measles virus [83].

The mode of cell death matters mechanistically. Vesicular stomatitis virus (VSV)-induced pyroptosis via Gasdermin E (GSDME), rather than apoptosis, was shown to be necessary for cytotoxic T-lymphocyte recruitment and for sensitizing cold tumors to checkpoint blockade. In result, depletion of GSDME abrogated both the antitumor immune activation and the sensitization effect [84]. This pathway is not unique to VSV. Oncolytic parapoxvirus similarly induces GSDME-dependent pyroptosis, stabilizing GSDME through decreased ubiquitination in GSDME-low tumor cells, with GSDME depletion again reducing intratumoral cytotoxic T-lymphocyte infiltration and abolishing checkpoint sensitization [85]. Notably, only a small fraction of tumor cells need undergo pyroptosis to drive broader immune infiltration and systemic antitumor immunity, which helps to explain why partial or heterogeneous OV infection can still convert an immune-desert or immune-excluded tumor into an immune-inflamed one [86,87]. However GSDME is broadly expressed in normal tissues, so unconstrained pyroptosis induction carries a toxicity risk, motivating engineered platforms that spatially and temporally restrict gasdermin activation, for example inducible gasdermin payloads confined by tumor-targeted delivery systems [88,89].

If these inflammatory perturbations result solely in antiviral-dominant inflammation without effective tumor-antigen cross-presentation and functional antitumor T-cell responses, therapy stalls at non-productive immune activation (Figure 1A). According to the interpretive hierarchy of on-treatment evidence, intermediate inflammatory remodeling (marked by IFN, CD8, and PD-L1 signals) must translate into functional tumor recognition and spatial cytotoxicity to confirm true productive immune conversion (Figure 1A, bottom).

#### 6.1.3. Post-Conversion Immune States and Checkpoint-Amplifiable Response

When preserved antigen presentation, effective T-cell infiltration, and functional cytotoxic contact with viable tumor cells occur without dominant immunosuppressive barriers, tumors enter an effector-permissive state, harboring the intrinsic potential for OV-driven tumor control even in the absence of checkpoint blockade (Figure 1B).

More commonly, active antitumor immunity is constrained within a checkpoint-restrained state, where tumor-reactive T cells reach viable malignant tissue and retain intact antigen recognition, but reversible checkpoint signaling becomes the dominant limitation (Figure 1B). The immune context generated by increased tumor-infiltrating lymphocytes, upregulated PD-L1 and interferon-driven gene expression signatures creates the substrate on which checkpoint blockade can act, since immune checkpoint inhibitors (ICIs) are inherently limited to tumors with adequate lymphocytic infiltration [90,91,92]. This is illustrated clinically by coxsackievirus A21 plus pembrolizumab in the CAPRA trial, achieving a 47% objective response rate in melanoma, exceeding either monotherapy, with a median progression-free survival (PFS) of 11.9 months, median overall survival (OS) of 30.9 months and 82% of responses lasting ≥6 months [31,38,93,94]. Notably, response in CAPRA did not correlate with baseline tumor PD-L1 expression, ICAM-1 expression, or CD3+ infiltrate. Baseline CD3+CD8− T-cell density was in fact significantly lower among responders. Instead, response tracked with on-treatment increases in serum CXCL10 and CCL22, suggesting that the intensity of viral replication and infection, rather than a pre-existing hot microenvironment, was the operative driver of checkpoint-amplifiable response [93,94]. This complicates the assumption that OV-checkpoint synergy depends strictly on baseline immune infiltration and instead points to on-treatment immune conversion as the more relevant biomarker axis [95].

A 2025 systematic review and meta-analysis of 36 trials, including approximately 4190 patients, has found that OV-based regimens roughly tripled objective response rate and improved PFS and OS versus standard therapy without excess grade ≥3 toxicity. Response was associated with high tumor mutational burden, interferon-pathway loss-of-function mutations, baseline CD8+ infiltration, post-OV IFN-γ/PD-L1 upregulation, and favorable gut microbial signatures, while intact antiviral signaling, immune-excluded microenvironments, and myeloid dominance predicted resistance [96]. In these settings, converting biological rationale into clinical efficacy requires therapeutic strategies that directly target the dominant non-checkpoint barrier.

### 6.2. Why Immune Activation May Be Insufficient

Immune activation initiated by oncolytic viruses (OVs) does not guarantee a durable antitumor response. Failure can occur at the level of viral delivery, at the level of immune conversion itself, or downstream through suppressive and spatial barriers that re-exclude effector cells from the tumor [97].

#### 6.2.1. Failure of Viral Delivery/Infection

Despite advances in the field, delivery remains a central bottleneck and the choice between local and systemic administration involves distinct trade-offs. Tumor-associated stroma, including dense extracellular matrix components such as hyaluronic acid, forms a physical barrier that impedes viral penetration and spread, limiting therapeutic efficacy regardless of route [95,98,99].

Intratumoral administration achieves high local viral concentration and stimulates a localized immune response capable of affecting neighboring, non-injected lesions, but it is constrained by limited accessibility of deep-seated or disseminated disease [71,100,101]. Intravascular delivery aims to distribute OVs throughout the body, in principle reaching both primary and metastatic lesions, but its efficiency is limited by rapid clearance by the mononuclear phagocyte system, neutralization by pre-existing and treatment-induced antibodies, off-target sequestration, and physical barriers within the tumor itself. Pre-existing humoral immunity against common OV platforms, such as the previously discussed adenovirus type 5, HSV-1, vaccinia, measles or reovirus, is prevalent in the general population, and growing evidence suggests neutralizing antibodies affect not only viral infection and spread but also the character of the induced immune response, with effects that are not uniformly inhibitory and depend on route of administration, tumor vascularization, and viral mechanism of action [102,103]. As a result, intravenous administration may require doses 10- to 100-fold higher than intratumoral injection to reach comparable tumor levels, raising the potential for toxicity. Strategies such as receptor retargeting, cell carriers, polymer coating, and liposomal encapsulation have been explored to shield virions from immune clearance during systemic transit [68,69,104].

#### 6.2.2. Failure of Antitumor Immune Conversion

Even when infection is achieved, the balance between antiviral and antitumor immunity determines whether productive immune conversion follows. Single-agent OV administration limits biosafety concerns and regimen complexity, but the virus must persist long enough and induce sufficient oncolysis to generate durable antitumor immunity. Because host immunity can eliminate the virus before this threshold is reached, a single administration may produce only a low or moderate effect, and advanced-stage disease may require repeated dosing, which increases the risk of neutralizing antibody development [104].

Tumor-associated macrophages (TAMs) represent an important, specific mechanism of failed conversion. TAM infiltration correlates with resistance to oncolytic virus M1 across multiple syngeneic solid tumor models, and TAM depletion increases intratumoral viral load and GZMB+ CD8+ T-cell accumulation, with the benefit abrogated by CD8+ T-cell depletion, confirming TAMs actively suppress viral persistence and T-cell-mediated conversion [71].

#### 6.2.3. Failure Caused by Suppressive or Spatial Barriers

Immune conversion can also be actively reversed by recruitment of suppressive populations into the tumor microenvironment. Oncolytic virus M1 induces tumor-associated myeloid cells to infiltrate tumors via an IL-6/PI3K-γ/Akt axis, and these tumor-associated myeloid cells directly quench CD8+ T-lymphocyte activity; pharmacologic PI3K-γ inhibition relieves this suppression and restores OVM efficacy, particularly when combined with checkpoint blockade [71]. Notably, the relationship between OVs and myeloid-derived suppressor cells (MDSCs) is not uniformly antagonistic to therapy. Oncolytic reovirus has been shown to reduce MDSC-mediated suppression of CD8+ T-cell proliferation in a TLR3-dependent, but not IFN-β promoter stimulator-1-dependent, manner, indicating that the net effect on myeloid suppression varies by virus, timing, and tumor context [68,69].

While oncolytic virotherapy generally modulates the immune microenvironment favorably, regulatory T cells (Tregs) can actively adapt to form an additional functional barrier. For instance, rather than decreasing immunosuppression, systemic administration of oncolytic virus M1 increases intratumoral Tregs with elevated CD25 expression and enhanced suppressive activity on CD8+ T cells. Overcoming this virus-induced barrier requires specific combination strategies. Treg depletion, or specific targeting with anti-CTLA-4 antibody combined with M1, significantly improves antitumor effect, increasing CD45+, CD8+, and CD4+ T-cell infiltration while decreasing the Treg-to-CD4 ratio [104]. Similarly, combining Newcastle disease virus or oncolytic HSV-1 with anti-CTLA-4 antibodies increases the ratio of effector T cells to Tregs and recruits more effector T cells into the tumor while reducing Tregs [69].

Finally, translation from preclinical models to clinical application remains inherently limited: animal models rarely replicate the full stromal, immunologic, and heterogeneous complexity of human tumors, constraining the predictive value of preclinical resistance and efficacy data [104].

### 6.3. Arming Oncolytic Viruses with Therapeutic Transgenes

A defining advantage of OVs over most other anticancer modalities is that they are genetically tractable delivery vehicles. The viral genome can accommodate one or more exogenous transgenes that are expressed preferentially within the tumor as the virus replicates. In principle the number of payloads that can be inserted is almost unlimited, and the field has consequently produced a large and heterogenous body of “armed” constructs [1,105,106]. The core rationale for arming is twofold. First, in-tumor transgene expression concentrates a biologically active payload at the site of disease while limiting the systemic exposure that would accompany the same molecule given intravenously. Second, arming converts the virus from a purely lytic agent into a multifunctional immunotherapeutic that can simultaneously lyse tumor cell and reshape the surrounding microenvironment [54,107].

#### 6.3.1. Immunostimulatory Cytokine Payloads

The most clinically mature arming strategy is the insertion of immunostimulatory cytokines. The prototype is talimogene laherparepvec (T-VEC), a herpes simplex virus type 1 engineered to express granulocyte-macrophage colony-stimulating factor (GM-CSF) to promote recruitment and maturation of antigen-presenting cells at the lysis site [1]. Subsequent constructs have targeted more potent T-cell-polarizing cytokines. An interleukin-12-encoding VSV-GP enhanced antitumor efficacy by inducing CD8+ T-cell responses with a durable, long-lived effector phenotype [14], and cytokine-armed adenoviruses increased intratumoral T-cell recruitment in renal cell carcinoma models [56]. Because systemic exposure to IL-12 is dose-limiting, a central engineering theme is restricting payload activity to the tumor. A tumor-conditional IL-15, expressed only under tumor-restricted control, synergized with immunotherapy while limiting the toxicity associated with constructive cytokine expression [62].

#### 6.3.2. Bispecific T-Cell Engagers

Rather than relying solely on endogenous priming, some constructs encode bispecific T-cell engagers (BiTEs) that are secreted locally and physically crosslink T cells to target cells. An OV expressing a T-cell engager was shown to target cancer cells and immunosuppressive stromal cells simultaneously [53], and an oncolytic adenovirus secreting a fibroblast activation protein (FAP)-targeted BiTE redirected T cells against the tumor stroma [67]. Locally restricted BiTE expression is attractive precisely because systemically administered engagers are limited by on-target off-tumor toxicity.

#### 6.3.3. Stromal and Extracellular Matrix-Remodeling Payloads

Dense stroma and extracellular matrix constitute a physical barrier to both viral spread and effector-cell infiltration. Transgenes encoding matrix-degrading enzymes address this directly: an oncolytic vaccina virus encoding hyaluronidase reshaped the extracellular matrix and enhanced the activity of co-administered chemotherapy and immunotherapy [15]. The FAP-targeted BiTE construct above also functions in part as a stroma-directed strategy [67], illustrating that a single payload can serve more than one functional category.

#### 6.3.4. Payloads That Amplify Immunogenic Cell Death

Because the immunogenicity of oncolysis depends on the mode of tumor-cell death, transgenes can be used to bias cells toward highly immunogenic death programs. Genetic engineering of systematically injectable OVs to accelerate pyroptosis enhanced antitumor activity. Moreover these platforms were designed to confine gasdermin-driven pyroptosis to the tumor to avoid the toxicity that would accompany unrestricted activation in normal tissue [88].

#### 6.3.5. Tumor Antigen and Neoantigen Payloads (In Situ Vaccination)

Arming can also supply the antigenic cargo itself, turning the virus into a personalized vaccine platform. Neoantigen-encoded OVs deliver patient-specific tumor antigens directly into the inflammatory context created by oncolysis, combining adjuvant and antigen in a single agent [7]. Earlier armed replicating vectors, such as a novel armed ancolytic measles vaccine virus developed for cholangiocarcinoma, established the feasibility of expressing therapeutic transgenes from a replicating oncolytic backbone [37].

#### 6.3.6. Shielding and Delivery-Enabling Modifications

A final category addresses the virus’ own vulnerability to host clearance rather than the tumor. Displaying complement-regulatory proteins on the viral surface, for example a CD55-displaying oncolytic vaccinia virus, allowed the virus to evade host innate and adaptive humoral responses and treat metastatic disease, extending the reach of systemic delivery [108].

### 6.4. A Simplified Response Model for OV-Based Therapy

Whereas OV-mediated immune conversion is an ongoing biological process, this review formulates a simplified translational model that divides the ongoing process into eight interrelated steps. The eight-step model is deliberately designed to represent bottlenecks met in therapy, from delivery to the clinical endpoint, where a biological correlation of each step can be used in therapy. Table 3 summarizes the eight translational steps in OV activity.

## 7. Checkpoint Inhibitor Synergy: Rationale and Clinical Evidence

### 7.1. Biological Rationale for OV–ICI Combinations

The primary goal of combining oncolytic viruses (OVs) with immune checkpoint inhibitors (ICIs) is to overcome the limitations of single-agent immunotherapy [117]. The main mechanism of action of ICIs is the reactivation of pre-existing anti-tumor immunity, which is why they usually do not work in immune-desert and immune-excluded tumors. This is due to their low T-cell infiltration, dense extracellular matrix, and low mutational burden [31,91]. The detailed mechanisms behind converting cold tumors into inflamed phenotypes, along with the action of immunostimulatory signals, are thoroughly described in Section 5 and Section 4.2, respectively.

In many models and early clinical studies, OV-induced inflammation is accompanied by upregulation of PD-L1 on cancer cells and tumor-associated immune cells. Processes that drive this overexpression are the release of proinflammatory cytokines, IFN signaling, and local IFN-γ accumulation. This upregulation driven by inflammation creates adaptive immune resistance, simultaneously recruiting T cells and triggering checkpoint ligands to suppress them [31,91,106,109,110,111,112,113,114,115,116,117,118,119,120,121,122,123].

Anti-PD-1/PD-L1 and anti-CTLA-4 antibodies act synergistically by effectively blocking the reactivated PD-1/PD-L1 axis to reverse T-cell exhaustion and restore the proliferative and cytotoxic functions of CD8+ lymphocytes. By sustaining the antitumor response initiated by OVs, checkpoint inhibitors foster the generation of long-lived memory CD8+ T cells and can enable the systemic destruction of distant, non-injected metastases through the abscopal effect [31,106,117].

### 7.2. Clinical Evidence for OV–ICI Combinations

Currently, the most conclusive evidence regarding the efficacy of OV-ICI combinations concerns melanoma, particularly regimens based on T-VEC and RP1. In the case of other viruses and other tumor types, information comes from earlier-phase studies, was conducted on smaller patient cohorts, or remains inconsistent [31,117,120,124]. Key clinical trials and representative strategies evaluating OV–ICI combinations across various tumor types are summarized in Table 4.

In a Phase II randomized study from 2023, Chesney et al. demonstrated superior efficacy of Talimogene laherparepvec (T-VEC) with ipilimumab (anti-CTLA-4) over ipilimumab monotherapy in patients with unresectable stage IIIB–IV melanoma. In the primary analysis of the randomized Phase II trial, initial objective response rates (ORRs) were reported at 39% versus 18%. In the final 5-year mature analysis, T-VEC plus ipilimumab maintained significantly higher ORR (35.7% vs. 16.0%) and durable response rates (33.7% vs. 13.0%) compared to ipilimumab alone [120]. The median duration of response was 69.2 months in responding patients, and the estimated 5-year overall survival (OS) reached 54.7% vs. 48.4%. The safety profile was acceptable and showed no new toxicity signals in long-term follow-up. Responses were observed in injected sites as well as in distant visceral metastases, confirming the abscopal effect [117,120].

The combination of T-VEC with pembrolizumab (anti-PD-1), in the Phase Ib portion of the MASTERKEY-265 study, a very high ORR of 67% (with 43% complete responses, CR) and a 3-year OS of 71% in 21 previously untreated melanoma patients [119,125]. However, the subsequent randomized Phase III study did not meet its primary endpoints since the addition of T-VEC to pembrolizumab did not significantly improve progression-free survival (PFS; HR 0.86, 95% CI 0.71–1.04) or OS (HR 0.96, 95% CI 0.76–1.22) relative to pembrolizumab plus placebo [120].

In the IGNYTE study in patients with advanced melanoma with progression after prior anti-PD-1 treatment, the combination of GAL-1/RP1 (vusolimogene oderparepvec) with nivolumab demonstrated high clinical activity, with an ORR of 32.9–33.6% and a CR of 15.0–16.4%. In turn, the median duration of response was 24.8–33.7 months, and the 2-year OS reached 63.3% with low toxicity, as grade 3/4 adverse events occurred in 12.9% of patients. Randomized Phase III evaluation (IGNYTE-3) is currently ongoing to confirm these findings against physician’s choice therapy [121].

In a Phase II study from 2019, CAPTIVE/KEYNOTE-192, in patients with recurrent glioblastoma, a median OS of 12.5 months and disease control in 56.2% of treated patients were achieved following the administration of DNX-2401 with pembrolizumab, which was associated with virus-induced intense infiltration of CD8+ lymphocytes [116].

Bladder cancer (CVA21) proved to be a promising model in a Phase I study on Coxsackievirus A21 (CAVATAK). It was demonstrated that the virus replicates efficiently in tumor tissue and induces local inflammation within it. It also increased the expression of PD-L1 and interferon-activated genes without causing significant toxicity, providing a strong biological rationale for combining this therapy with other drugs [122].

In a Phase I trial published in 2025, MV-NIS was used to treat children and young adults with recurrent atypical teratoid/rhabdoid tumor (AT/RT) or medulloblastoma. The study enrolled 34 patients, of whom 23 with medulloblastoma and 4 with AT/RT were evaluable. The findings indicated a potential immunological effect of the therapy, although their significance was limited by the small study group [36,126].

### 7.3. Why Clinical Results Remain Inconsistent

A major issue with combining oncolytic viruses (OVs) and immune checkpoint inhibitors (ICIs) is the high variability of results, which is driven by multiple factors.

One of the primary sources of inconsistent clinical trial outcomes is the lack of biomarker-driven patient selection. Clinical trials have typically enrolled patients based on tumor type and anatomical accessibility for treatment. Unfortunately, key variables such as tumor permissiveness to viral infection, antiviral status, and immune contexture have been neglected, despite growing evidence demonstrating that therapeutic success strongly correlates with these parameters [127,128,129]. There is also a lack of easily measurable biomarkers.

The consequences of this are evident in clinical settings. For instance, in a 2019 trial evaluating DNX-2401 in combination with pembrolizumab for recurrent glioblastoma, the primary efficacy endpoint was generally not met. However, exploratory analyses revealed that objective responses were observed exclusively in tumors with an intermediate immune status [116].

Another key factor contributing to inconsistent treatment outcomes is profound inter- and intratumoral heterogeneity. Tumors vary significantly in terms of their susceptibility to viral infection, viral replication capacity, stromal density, vascular permeability, antigenicity, and immune cell infiltration. Consequently, the same OV–ICI regimen can result in vastly different levels of viral amplification and immune system activation across individual patients [117,127].

It is worth noting that the suppressive architecture of the tumor microenvironment (TME) also contributes to this heterogeneity. Myeloid-rich, fibrotic, or metabolically hostile tumors can remain resistant to therapy even after viral infection. This occurs because blocking the PD-1/PD-L1 or CTLA-4 pathways addresses only a fraction of the immunosuppressive network. Compensatory pathways such as T-cell immunoreceptor with immunoglobulin and ITIM domains, lymphocyte-activation gene 3, T-cell immunoglobulin and mucin-domain containing 3, VISTA, adenosine, or indoleamine 2,3-dioxygenase (IDO) can maintain immune dysfunction despite ongoing viral therapy [117,130].

In addition, viral delivery to the tumor interior is not uniform, creating further obstacles to achieving consistent study results. Direct intratumoral administration achieves high local concentrations, but it is feasible only in select cases and fails to ensure homogeneous distribution throughout large, fibrotic, necrotic, or poorly vascularized tumors [107,127].

## 8. Biomarker-Guided Clinical Translation

### 8.1. Baseline Biomarkers for Patient Selection

The baseline biomarkers are unique factors, which can be used to qualify patients for OV therapy. They have been classified as the following: (1) viral entry receptor expression, (2) interferon signaling pathway, (3) immune profile and (4) genomic features of tumors. None of these markers are consistent predictors in all types of cancers and all oncolytic viruses used in clinical trials [19,96].

#### 8.1.1. Viral Entry Receptor Expression

Presence of the transmembrane protein adipocyte-associated 1 (TPRA1) has been established as a critical biomarker of the predictability of the therapeutic efficacy of the oncolytic virus M1 (OVM). TPRA1 acts as a receptor of the virus with its glycosylated extracellular domain mediating interaction with the virus and with its C-terminal cytoplasmic domain contributing to the process of viral internalization. Expression of TPRA1 has been associated with increased sensitivity to the OVM treatment in different cancer cells, mouse tumor models, and patient-derived bladder and prostate cancer cells [20]. The significance of the existence of viral receptors in predicting the effectiveness of the OVM treatment has also been confirmed by identification of another viral receptor—MXRA8. However, just the presence of viral receptors is not enough to predict the effectiveness of the treatment as the state of tumor cells concerning their intracellular antiviral activity is equally important; specifically, low levels of the antiviral zinc-finger antiviral protein (ZAP) have been found to correlate with increased OVM effectiveness. Importantly, the combination of MXRA8 and ZAP is a more effective predictive marker of OVM effectiveness than any of these factors alone [19]. In a similar way, the baseline tumor biomarkers indicating viral permissibility can be used as the basis for selection of the patients for Seneca Valley virus (SVV-001) treatment. The cellular receptor of the virus, TEM8/ANTXR1 (anthrax toxin receptor 1), is therefore a potentially important biomarker of patients’ likelihood to benefit from the treatment. Nonetheless, the presence of TEM8/ANTXR1 is apparently not enough for infection with the SVV-001 as the viral permissibility correlates with reduced expression of the genes that are part of type I interferon antiviral signaling pathway. Specifically, the SCLC-N subtype characterized by the elevated levels of NEUROD1 and low levels of ASCL1 has been associated with SVV-001 permissivity in small cell lung carcinoma (SCLC) [21,131].

#### 8.1.2. Interferon Signaling Pathway

**Figure 3:**
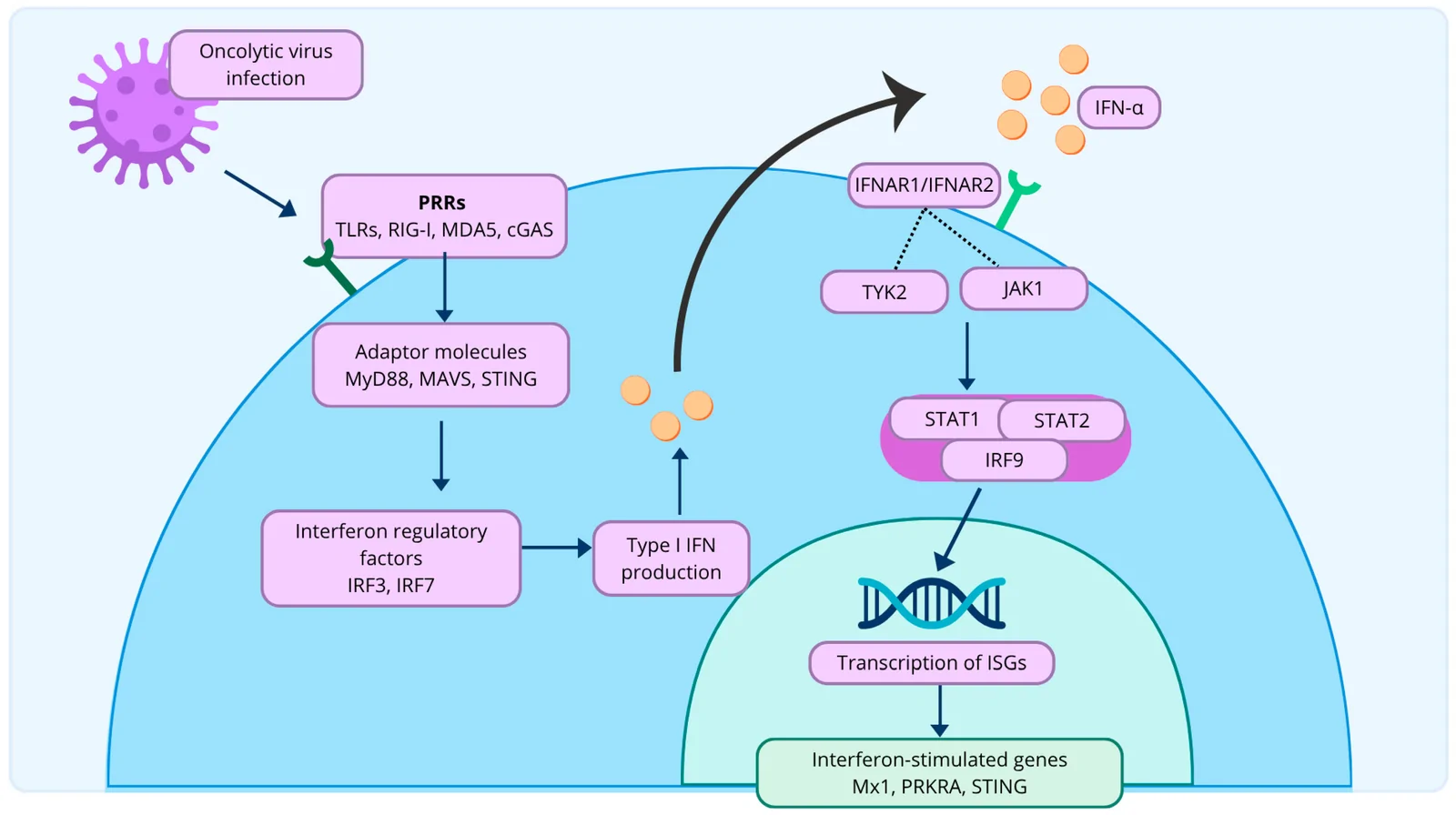
Simplified scheme of the type I interferon (IFN) signaling pathway upon oncolytic virus infection. Following cell entry, viral components are detected by intracellular pattern recognition receptors (PRRs: TLRs, RIG-I, MDA5, cGAS), recruiting adaptor molecules (MyD88, MAVS, STING) to activate IRF3/IRF7 and induce Type I IFN (e.g., IFN-α) production. Secreted IFN-α binds to the IFNAR1/IFNAR2 receptor complex, activating receptor-bound kinases (TYK2 and JAK1). This triggers the formation of the STAT1/STAT2/IRF9 complex, which translocates to the nucleus to induce the transcription of interferon-stimulated genes (ISGs, such as Mx1, PRKRA, and STING).

The IFN signaling pathway represents another good candidate for biomarker in OV treatment due to the possible increased susceptibility of cancer cells with defective IFN induction or response to viral replication and oncolysis. Components of the pathway include various pattern recognition receptors (PRRs), adaptor molecules, interferon regulatory factors, IFN receptors, the JAK/STAT pathway and interferon-stimulated genes (ISGs). PRRs include TLRs, RIG-I-like receptors (RLRs), comprising retinoic acid-inducible gene I (RIG-I) and melanoma differentiation-associated protein 5 (MDA5), and cyclic GMP–AMP synthase (cGAS). These receptors initiate distinct downstream signaling pathways. TLRs signal primarily through the adaptor proteins MYD88. RIG-I and MDA5 signal through mitochondrial antiviral-signaling protein (MAVS). cGAS activates stimulator of interferon genes (STING) through the production of cyclic GMP–AMP (cGAMP). These pathways converge on the activation of interferon regulatory factors 3 and 7 (IRF3 and IRF7), promoting the production of type I interferons, including IFN-α and IFN-β. Then it binds to the heterodimeric interferon-α/β receptor (IFNAR), composed of the IFNAR1 and IFNAR2 subunits. This triggers the activation of JAK1 and tyrosine kinase 2 (TYK2). These kinases phosphorylate STAT1 and STAT2, which subsequently associate with interferon regulatory factor 9 (IRF9) to form the interferon-stimulated gene factor 3 (ISGF3) complex. ISGF3 then translocates to the nucleus, where it binds to interferon-stimulated response elements (ISREs) in target gene promoters and induces the transcription of interferon-stimulated genes (ISGs), thereby establishing an antiviral cellular state. This process is presented in a simplified schematic in Figure 3.

Mutations and dysregulation of expression in the components of this pathway have been identified as predictors of sensitivity to various oncolytic viruses. However, constitutive activation of the IFN pathway and ISG was associated with resistance to the oncolytic measles virus and a gene-expression-based diagonal linear discriminant analysis model was generated to predict treatment response. MYD88 expression was significantly higher in cultures of breast cancer patients resistant to oncolytic HSV-1 therapy and several other ISGs (Mx1, PRKRA, STING) showed potentially high predictive accuracy regardless of the lack of statistical power of the analysis. Type I IFN responses differences in tumor cell lines have been found to correlate with resistance to VSV, while EGFR/HER2 signaling has been put forward as a contributing factor in this correlation and the latter protein has been suggested as a possible marker of resistance. Thus, an evaluation of mutations or alterations in the pathway together with some IFN-related gene signatures may be a tool for prediction of response to treatment with certain OVs [78,79,132].

#### 8.1.3. Immune Profile

Immune profiling based on peripheral immune cells could represent a non-invasive strategy for determining which patients might respond better to OV treatment. Patients who received TILT-123 showed an increased baseline presence of CD16+ monocytes, reduced levels of regulatory T cells (Tregs), and an enhanced cytotoxic transcriptional profile including genes such as CD8A, NKG7, GNLY, GZMB, and PRF1. Similarly, responders to Pexa-Vec had an immune system less prone to exhaustion, based on their reduced baseline levels of Tregs and PD-L1 expression in peripheral immune cell populations, while increased Tregs levels and immune-checkpoint expression represented hallmarks of an exhausted immune phenotype. Overall, these findings suggest that analysis of peripheral immune cell composition, cytotoxic properties, immune checkpoint expression, and specific transcriptional profiles might be used for selecting patients with a favorable baseline immune profile for OV treatment [133,134].

#### 8.1.4. Genomic Features of Tumors

Genomic alterations in tumors may have a significant effect on the effectiveness of OVT by affecting tumor cells’ susceptibility, viral replication, and antitumor immunity. For example, clinically relevant genomic alterations in ovarian cancer, including TP53, BRCA1/2, PTEN, NF1, and KRAS mutations, have been associated with heterogeneous responses to oncolytic viruses. Notably, KRAS G12V mutants were shown to increase sensitivity to VSVΔM51, and VSVΔM51 increased survival in models with PTEN/KRAS, PTEN/TP53, and TP53/BRCA2 mutations and enhanced CD4+ and CD8+ T-cell activation [135]. On the other hand, the loss of the host gene IP6K2 decreased HSV-1 replication and virus-mediated apoptosis, causing resistance to HSV-1 oncolysis. Thus, the presence of the R212H/C variants in the IP6K2 gene may be considered as a potential genomic alteration affecting the efficiency of HSV-1 treatment, although its causative role has not been proven yet [136]. Another example is the SH2B3 P521L variant found only in recurrent lesions of Merkel cell carcinoma that were described as resistant to the treatment with talimogene laherparepvec (T-VEC); increased variant allele frequency corresponded to increased interferon-α and inflammatory pathways activation [137].

#### 8.1.5. Antiviral–Antitumor Immunity Trade-Off

Oncolytic virus treatment creates a complex interplay between antiviral and antitumor immune response, where increased immune activation can enhance tumor recognition as well as prevent viral persistence. The destruction of tumor cells through OVs and subsequent release of viral and tumor-associated antigens, as well as DAMPs and PAMPs, induce both innate and adaptive immunity and could contribute to converting a cold tumor into an inflamed and immune-permissive environment. More specifically, type I interferon signaling is involved in inflammatory activation, antigen presentation, and immune effector cells recruitment, thus providing the development of antitumor immunity. However, at the same time, antiviral IFN-dependent responses inhibit viral replication and contribute to early virus elimination and hence restrict OV-based therapeutic effect [95,138]. It should be noted that an intense inflammatory antiviral response is not a guarantee for developing tumor-specific immunity because of the large number of viral antigens and cytokines which can drive T-cell response to antiviral rather than antitumor clones; in addition, type I IFN-mediated mechanisms can also affect the loss and suppression of antitumor T-cell function [139]. Therefore, the therapeutic efficiency of immune activation seems to depend on finding the balance, when antiviral responses are enough to change tumor microenvironment and recruit antitumor immunity without overwhelming antiviral immunodominance and restrictions of OV replication via IFNs [95,138,139].

### 8.2. On-Treatment Biomarkers of Response During OV Therapy

The in-vivo markers for OV response include the induction of interferon post-therapy, release of cytokines and chemokines, proliferation of viral specific T cells, changes in lymphocyte subsets in the periphery, and fluctuating amounts of viral DNA in the bloodstream. These parameters are evaluated early in the treatment process to determine the presence of an effective anti-tumor immune response [140,141].

#### 8.2.1. PD-L1 and Interferon Signaling

Immunological changes during treatment have been shown to be correlated with the therapeutic effect from OV treatment and can distinguish between responders and non-responders. In patients responsive to Pexa-Vec treatment, there was induction of interferon response with increased production of inflammatory cytokines and chemokines, including CXCL10, CXCL9, CCL4, CCL7, and IL-16, and such alterations were related to the activation and migration of immune cells. Moreover, induction of upregulation of PD-L1 after the treatment was more frequent among the responders and was related to the inflammatory, IFN-mediated antitumor response, while the pre-existing expression of PD-L1 was indicative of chronic exhaustion. The induction of increased number of CD8+ T cells infiltration, PD-L1 expression, and IFN-γ gene expression was also observed after treatment in patients responsive to T-VEC in combination with anti-PD-1, while no correlation between the basal levels of CD8+ T cells and IFN-γ expression signature and response was found. By contrast, in non-responders, higher levels of various cytokines and chemokines were found, however, without post-treatment induction as in responders [96,125,134].

#### 8.2.2. Viral DNA and OV-Specific T-Cell Dynamics

The presence of persistent viral DNA in the peripheral blood was used to determine the level of in vivo viral amplification in terms of different NK- and T-cell responses based on the presence of either transient or persistent viral DNA in the blood. The responses of cytokines, T-cells, and NK cells were determined to occur as soon as one day after the administration of oncolytic vaccinia virus. Peak levels of the responses occurred at about a week and continued for about a month. In another clinical study, the presence of OV-specific IFN-γ producing T cells was found in peripheral blood, and more patients exhibited OV-specific T cell responses after expanding the T-cells for 10 days ex vivo. The OV-specific T cell responses were related to initial clinical response while the responses of cytomegalovirus, Epstein–Barr virus, and influenza peptide stimulations were not related to the clinical response [140,141,142].

#### 8.2.3. Immune Activation as a Biomarker of Response to OV Therapy

In addition to oncolytic properties, OVs can increase the ability of the tumor microenvironment to present antigens and facilitate the formation of a functional antitumor immune response. Interferon type I produced as a result of OV infection helps in the upregulation of MHC class I and II molecules, as well as of costimulatory molecules such as CD40, CD80, and CD86. This helps in the processing and presentation of tumor antigens, and also in the activation of tumor-specific T-cells. Thus, OVs can improve CD8+ T cell activation and cause not only local regression of the infected tumor, but also that of distant tumors, due to the systemic nature of the antitumor immune response. In this case, the OV-based therapy can be considered as a form of in situ vaccination when tumor antigens, produced after viral oncolysis, are used to initiate antitumor immune response [98,106,143].

### 8.3. Biomarkers of Resistance During OV Therapy

Resistance to oncolytic virotherapy is determined by a complex multifactorial environment involving tumor cell intrinsics, immune, and microenvironmental parameters, and there is currently no biomarker that can effectively predict responsiveness [78]. However, we can distinguish couple of mechanisms, that take part in this problem, like activation of IFN axis, specific TME and defined immune cells [96,104,144,145,146,147].

#### 8.3.1. Interferon Axis as the Main Resistance Mechanism

The IFN response is one of the important factors of OV therapy resistance mechanisms. First of all, the ability of tumor cells to maintain functional type I IFN signaling pathway results in establishment of an antiviral state which in turn limits viral replication and oncolysis. The presence of elevated or persistent expression of interferon-stimulated genes (ISGs) such as MxA/Mx1 and OAS has been established in resistance to vesicular stomatitis virus (VSV) tumor cells. Moreover, secretion of IFN by OV-infected tumor cells activates JAK/STAT pathway in the neighboring tumor cells resulting in activation of antiviral proteins and additional inhibition of viral replication and oncolysis. Some ISGs including MxA have been found to be directly responsible for the restriction of VSV replication. As for the ovarian cancer models, it was shown that tumor cells able to produce type-I IFN develop an antiviral state after VSV infection [96,145,146].

#### 8.3.2. Tumor Microenvironment and Immune Biomarkers

Resistance to oncolytic virotherapy is greatly influenced by tumor microenvironment, which encompasses immune cells and stromal components affecting the viral administration, survival, and activity. For example, as we mentioned before in the Section 5.2.2, TAMs play a key role in the development of resistance during M1 OV therapy. In addition, the extracellular matrix (ECM) acts as a physical and biochemical obstacle to oncolytic virus (OV) delivery and spread due to secretion of collagen, hyaluronic acid, and other ECM components by cancer-associated fibroblasts. Such ECM condensation can lead to increased interstitial fluid pressure and compression of blood vessels, thus preventing the OV extravasation from tumor blood vessels and restricting interstitial diffusion [22,144].

Regulatory T cells (Tregs) that express FOXP3, characterized by CD4+CD25+Foxp3+ phenotype, form the primary suppressor cell type in the tumor microenvironment. The systemic administration of the oncolytic virus M1 resulted in Treg infiltration into the tumor microenvironment and enhancement of their suppressive function towards CD8+ T cells; depletion of Tregs, on the other hand, enhanced the effect of M1 on cancer. CD163 is an antigen expressed by M2c macrophages that exert anti-inflammatory functions through secretion of IL-10 and TGF-β. Moreover, there are macrophages expressing CD163 that are considered pro-tumorigenic and pro-angiogenic [104,145].

### 8.4. Tissue-Based and Spatial Pathology Assessment

The ability of spatial pathology provides an in situ assessment of the changes caused by the therapy in the tumor microenvironment without the loss of its spatial organization [148]. Classical immunohistochemistry was used for the detection of HSV-1 protein in post-T-VEC tissues, but there was no HSV signal in the post-treatment specimens examined, whereas the presence of small amounts of HSV-1 DNA was detected by quantitative real-time PCR [149]. Multiplex immunofluorescence combined with spatial image analysis allows identifying the phenotype and spatial distribution of immune cells in tumor tissue, as well as discrimination between tumor-enriched and tumor non-enriched regions [148,149].

Spatial transcriptomics of pre- and post-T-VEC tissue specimens was performed with the help of NanoString GeoMx digital spatial profiling platform by selecting the regions of interest based on the fluorescent labeling of immune and stromal compartments. Analysis of the differences between central tumor tissue in pre- and post-T-VEC patients showed increased expression of transcriptional signatures related to CD8+ T cells in the post-treatment tissue. Multiplex immunofluorescence allowed demonstrating the heterogeneity of the localization of T cells, B cells, and macrophages in different tissue regions and groups of patients with different pathological responses [149]. An increase in the proportion of CD3+ and CD8+ T cells was observed in pre- and post-G207 treatment biopsy specimens, with the proportion of CD8+ cells increasing from 0.3% ± 0.1% to 3.8% ± 1.3%. In addition, tumor infiltration was observed in some regions of interest in post-G207 treatment tissue specimens, particularly in focal regions near the vascular niche. These findings were reported by Bernstock et al., who demonstrated a robust influx of CD3+ and CD8+ T cells following oncolytic herpes simplex virus G207 treatment, as well as robust tumor infiltration in focal regions of interest near the vascular niche approximately 90 days after treatment [150].

Comparison of pre- and post-treatment tissues also showed upregulation of CTLA-4 and PD-1 in lymphocytes, as well as PD-L1 and IDO. Upregulation of CTLA-4 and PD-1 occurred after treatment, whereas upregulation of PD-L1 and IDO was more frequent in post-treatment tissue [150].

OV_ICI combinations in selected cancers, together with candidates biomarkers for response to therapy, are presented in Table 5.

## 9. Delivery, Engineering, Safety Challenges and Adverse Effects

The preceding sections have focused on several challenges associated with effective tumor infection, productive immune conversion, and the induction of a sufficient antitumor immune response. All of these factors need to be adequately addressed to achieve the desired therapeutic effect. The route of administration of oncolytic viruses (OVs), including direct intratumoral injection, intravenous injection, intraperitoneal injection, and intracranial injection, may affect not only therapeutic efficacy but also patient safety, particularly with regard to treatment-related toxicity. This is especially relevant in immunocompromised patients, in whom systemic administration may potentially result in viremia. Particular concerns may arise following the intravenous administration of high doses of OVs. Another important safety consideration is viral shedding into the environment, which may potentially result in unintended exposure or infection of susceptible individuals.

Considering these challenges, an optimal OV-based therapeutic agent should ideally combine high tumor selectivity with limited or absent replication in healthy tissues, potent antitumor activity, and minimal viral shedding following administration. Recent advances in the genetic engineering of selected viruses have provided promising opportunities to develop such next-generation OV-based therapeutics. For example, preclinical studies of herpes simplex virus type 1 (HSV-1) and the Edmonston strain of measles virus (MeV) attenuated vaccine have demonstrated promising safety profiles, with low levels of treatment-related toxicity. Preclinical studies have also reported limited or undetectable viral shedding following administration, suggesting that viral dissemination may remain largely restricted to the sites of administration [155].

An increasing number of genetic engineering strategies are being investigated to enhance the therapeutic efficacy and safety of OVs. Next-generation OV-based therapeutics may offer improved safety profiles compared with earlier generations of these agents. Nevertheless, viral shedding and the potential for spontaneous recombination involving genetically modified oncolytic viruses remain important considerations that warrant further investigation. In our view, greater emphasis on the environmental safety assessment of novel OV-based therapeutics may facilitate their regulatory evaluation and clinical translation by regulatory agencies, including the U.S. Food and Drug Administration (FDA) and the European Medicines Agency. Such studies may also help minimize potential environmental risks associated with the release of genetically modified OVs following clinical administration [156,157].

Oncolytic viruses are designed to selectively infect and destroy malignant cells; however, their administration can result in a range of adverse effects. Several factors influence the safety profile of OV therapy, including the route of administration, viral platform, dose and characteristics of the treated tumor. Adverse effects can be broadly classified into local effects occurring near the site of drug administration and systemic effects arising from immune activation, treatment-related inflammation or viral dissemination. Most adverse events associated with oncolytic virotherapy have been reported to be mild to moderate; however, more severe systemic toxicities can occur in certain patients [158].

Local adverse events were more relevant when OV therapy was administered intratumorally and included injection-site pain, erythema, swelling tumor-associated inflammation. Intraperitoneal injection was well tolerated with adverse events limited to fatigue, malaise and abdominal pain. The most common systemic adverse effects associated with oncolytic virotherapy were nausea, fever, chills, pyrexia, vomiting, and headache. Common severe adverse events included neutropenia, anemia, lymphocytopenia, fatigue, leukopenia, transaminitis, thrombocytopenia, diarrhea and hypotension. Intravenous administration was associated with a higher incidence of severe adverse effects than intratumoral injection, intraperitoneal injection. While combining oncolytic virotherapy with other treatment methods increased therapeutic efficacy, it also resulted in higher toxicity risks compared to conventional methods. Notably, combination therapies including OV therapy and chemotherapy or radiotherapy significantly increased the risk of adverse events. The combination of OV therapy and immunotherapy resulted in a more favorable safety profile and clinical benefits. Efficacy varied across treatment strategies, administration routes, patient age groups (with adults exhibiting a more beneficial response to oncolytic virotherapy than pediatric patients) and virus classifications. Although adverse events were generally manageable, their occurrence highlights the importance of monitoring patients during oncolytic virotherapy for both local and systemic toxicity [158,159,160].

## 10. Future Perspectives

The future of oncolytic virotherapy depends on overcoming translational barriers associated with the immunosuppressive TME, the lack of immune cells in immune-desert tumors and stromal barriers in immune-excluded tumors, as well as improving delivery methods. One promising approach involves the use of viruses modified to express various transgenes that help counteract immunosuppression, such as TGF-β inhibitors or agents that degrade the ECM, thereby improving viral distribution. To date, this strategy has been primarily employed in vitro or in mouse models, where it has shown favorable results [15,50,60]. Therefore, further clinical validation is required.

Additionally, to avoid toxicity associated with systemic injection, an approach focused on transporting the virus into the TME may be beneficial. For instance, Eisenstein et al. proposed that in tumors where intratumoral injection is not feasible due to multifocal or inaccessible tumors, MDSCs could be used as vectors to deliver VSV to the tumor sites. They achieved encouraging results in mice [158]. However, the clinical efficacy of this strategy requires further investigation.

Furthermore, studies on combination OV-ICI therapy have provided inconsistent clinical results. Future research in this field should focus on standardizing immune biomarkers and individualizing therapy based on genomic profiles of tumors [128,129]. Greater attention should also be devoted to antiviral immunodominance. Overcoming this barrier by encoding tumor antigens into the viral genome represents a promising approach for promoting antitumoral T cell populations that can synergize with immune checkpoint blockade [161].

## 11. Conclusions

Current evidence in viral immunooncology indicates that the clinical efficacy of OVs does not stem from a single mechanism, but rather involves a complex cascade of biological processes. Direct oncolysis, although essential in the initial phase, primarily functions as a trigger for the release of signaling molecules, including DAMPs and PAMPs, along with the synthesis of type I interferons, which drives a fundamental shift in tissue immunogenicity. The observed synergy with ICIs relies on a complementary interaction. OV-mediated oncolysis and local inflammation can promote antitumor immune activation, whereas ICI can relieve checkpoint-mediated inhibition of T-cell function. A key insight derived from translational models is that predicting therapeutic response requires shifting away from static baseline parameters toward dynamic monitoring of on-treatment kinetics.

Despite a clear understanding of these core molecular pathways, several unresolved issues persist in the current field of study. First, significant biological heterogeneity remains a challenge due to the lack of standardized biomarkers to account for inter- and intratumoral variability. Second, the complex interplay and timing between host antiviral clearance and the generation of effective antitumor immunity remains insufficiently defined. Finally, spatial and stromal barriers present physical and metabolic microenvironmental hurdles that continue to impede viral spread and subsequent T-cell infiltration.

This information helps redefine the role of oncolytic viruses in modern oncology. It is clear that oncolytic viruses should no longer be evaluated merely as standalone cytolytic agents, but rather as biological tools for tumor phenotypic transformation. Furthermore, these findings carry critical implications for patient selection and trial design as special attention should be paid to patient stratification, moving beyond organ-specific tumor classification toward incorporating immunophenotypic characteristics and stromal architecture.

## Figures and Tables

**Table 1:** Summary of the Literature Search Strategy.

**Databases searched**	PubMed, Scopus, Web of Science, Google Scholar
**Search period**	January 2013–August 2026
**Final search date**	28 August 2026
**Language restrictions**	English
**Search terms**	“Oncolytic Viruses,” “Cancer Vaccines,” “Tumor Microenvironment,” “Immunogenic Cell Death,” “Immune Checkpoint Inhibitors,” “Biomarkers,” “Precision Medicine,” “Genetic Engineering,” “in situ cancer vaccination,” “antitumor immunity,” “biomarker-guided therapy,” and “engineered oncolytic viruses.”
**Initial records identified**	Approximately 600 records

**Table 3:** Simplified translational model of OV-induced immune conversion.

Translational Step	Observable Markers	Favorable vs. Unfavorable States	Potential Clinical Strategies	References
Viral delivery	Levels of intratumoral virus; titers of neutralizing antibodies; interstitial fluid pressure; stromal density	**Success**: Sufficient viral dose reaches tumor bypassing barriers **Failure**: Rapid antiviral clearance or physical barrier exclusion	Favor direct intratumoral injection; employ serotype switching or viral cloaking strategies for systemic delivery	[100,108,109,110,111]
Viral replication	Viral yield/titer within malignant tissue; constitutive IFN/ISG expression status	**Success**: Selective replication driven by oncogenic drivers or defective type I IFN **Failure**: Constitutive tumor-intrinsic antiviral resistance	Utilize IFN/ISG status as predictive biomarkers; apply JAK/STAT inhibitors to reverse viral resistance	[112]
Immunogenic oncolysis	Release of Calreticulin, ATP, and HMGB1; GSDME-dependent pyroptosis markers vs. silent apoptosis	**Success**: Induction of immunogenic cell death (ICD) and danger signal release **Failure**: GSDME loss blocking T-cell recruitment; potential off-target toxicity	Engineer OVs specifically for pyroptosis induction (e.g., STAT3 inhibitors) to safely boost the in situ vaccine effect	[84,85,86,87,88,89,113,114]
Innate immune activation	Local levels of type I IFNs and chemokines; density of CD103+ dendritic cells, NK cells, and macrophages	**Success**: PAMPs/DAMPs-driven immune cell recruitment and enhanced antigen cross-presentation **Failure:** Persistent immunosuppressive myeloid infiltrate	Combine OVs with myeloid-reprogramming agents, STING agonists, or ECM-degrading hyaluronidase	[112]
Adaptive immune response	Density of tumor-infiltrating CD8+ T cells; presence of tumor-antigen-specific memory T cells; epitope spreading	**Success**: Priming of tumor-reactive T cells generating durable memory **Failure**: Weak or absent T-cell priming leading to immune exhaustion	Validates the fundamental rationale for utilizing OVs as an in situ vaccine platform	[93,112]
Checkpoint relevance	On-treatment kinetics: magnitude of CD8+ infiltration and PD-L1 upregulation	**Success**: Conversion to a PD-L1-high, lymphocyte-infiltrated (“hot”) microenvironment **Failure**: Sole reliance on static baseline biomarkers fails to predict response	Use dynamic on-treatment immune kinetics to guide patient selection and precise timing of ICI combination	[93,112,115]
Resistance control	Viral propagation duration; NAb escalation rates; assessment of tumor mutational heterogeneity	**Success**: Overcoming early antiviral clearance to sustain biological effect **Failure:** Rapid viral elimination limiting efficacy against heterogeneous tumors	Implement cell-carriers, corona-replacement, or triggered viral platforms to sustain prolonged propagation	[88,90,108,109,111]
Clinical outcome	Survival metrics (OS, PFS, ORR); baseline CD8+; tumor mutational burden (TMB); post-treatment IFN-γ levels	**Success**: Objective tumor regression and measurable survival benefit **Failure**: Immune exclusion or myeloid dominance dictating clinical progression	Adopt biomarker-guided selection (mutational burden, ISG expression, NAb titers) specifically for OV-ICI combinations	[31,115,116]

Abbreviations: OVs: oncolytic viruses; NAb: neutralizing antibodies; IFN: interferon; ISG: interferon-stimulated gene; JAK: Janus kinase; STAT: signal transducer and activator of transcription; HMGB1: high-mobility group box 1; GDSME: gasdermin E; PAMPs: pathogen-associated molecular patterns; DAMPs: damage-associated molecular patterns; NK: natural killer; STING: stimulator of interferon genes; PD-L1: programmed death-ligand 1; INF-y: interferon gamma; ICI: immune checkpoint inhibitor.

**Table 4:** Selected clinical strategies combining oncolytic viruses with checkpoint inhibitors.

(a). Established OV-ICI combination trials																			
**Oncolytic Virus (OV)**	**Checkpoint Inhibitor (ICI)**		**Indication/Tumor Type**		**Trial Phase/Name**		**Study Design**		**Sample Size (N)**	**Comparator**		**Primary Endpoint**		**Major Efficacy Outcome**			**Evidence Status**		**References**
T-VEC (Talimogene laherparepvec)	Ipilimumab (anti-CTLA-4)		Unresectable Stage IIIB–IV Melanoma		Phase II (5-year final analysis)		Multicenter, randomized, open-label, two-arm		198 (combination N = 98; ipilimumab N = 100)	Ipilimumab alone		Investigator-assessed objective response rate (ORR) by immune-related response criteria		ORR 35.7% vs. 16.0% (OR 2.9, 95% CI 1.5–5.7, *p* = 0.0030; median duration of response 69.2 months vs. not reached; 5-year OS 54.7% vs. 48.4%			Established—randomized, primary endpoint met		[117,118]
T-VEC (Talimogene laherparepvec)	Pembrolizumab (anti-PD-1)		Advanced/Untreated Melanoma		Phase Ib & Phase III (MASTERKEY-265)		Phase Ib: single-arm, open-label; Phase III: randomized double-blind, placebo-controlled, global		Phase I b N = 21; Phase III N = 692 (346 per arm)	Placebo plus pembrolizumab		Dual primary endpoints: progression free survival (PFS) and overall survival (OS)		Phase Ib ORR 62%; Phase III ORR 48.6% vs. 41.3%, but PFS HR 0.86 (95% CI 0.71–1.04, *p* = 0.13) and OS HR 0.96 (95% CI 0.76–1.22, *p* = 0.74)—dual primary endpoints not met			Established—randomized phase III, primary endpoints not met despite numerically higher ORR		[119,120]
RP1 (Vusolimogene oderparepve)	Nivolumab (anti-PD-1)		Advanced Melanoma (anti–PD-1 resistant/failed)		Phase II (IGNYTE)		Single-arm, multicenter		140	None (single-arm)		Confirmed ORR by independent central review (RECIST v1.1)		ORR 32.9% (95% CI 25.2–41.3%; 15.0% complete response); median duration of response 33.7 months; 1- and 2-year OS 75.3% and 63.3%			Promising single-arm signal—basis for accelerated approval; confirmatory randomized data still pending		[121]
DNX-2401	Pembrolizumab (anti-PD-1)		Recurrent Glioblastoma		Phase II (CAPTIVE/KEYNOTE-192)		Single-arm, open-label, two-part (dose-escalation plus dose expansion combined)		49 enrolled (dose-escalation and dose-expansion combined)	None (single-arm; outcomes compared descriptively to a prespecified historical control rate)		Objective response rate per mRANO criteria (safety was the co-primary objective)		Primary ORR endpoint not statistically significant (~10% objective responses), but 12-month OS 53% versus a 20% historical control; a subset of responders had durable survival beyond 45 months			Early-phase/exploratory signal—not randomized, no concurrent comparator arm		[116]
(b). Platform or monotherapy studies evaluated for potential future ICI combination																			
**OV**		**ICI**		**Indication**		**Trial Phase**		**Study Design**			**Comparator**		**Primary Endpoint**		**Key Finding**	**Evidence Status**		**References**	
CVA21 (CAVATAK/Coxsackievirus A21)		N/A—OV monotherapy; evaluated for potential future ICI combination		Non-Muscle-Invasive Bladder Cancer		Phase I		Single-arm pre-surgical ICAM-1-targeted virotherapy window-of-opportunity study			None		Safety/feasibility and evidence of intratumoral viral activity		Intratumoral viral replication with immune cell infiltration, supporting mechanistic rationale for future ICI pairing	Exploratory/mechanistic—no ICI administered in this trial		[122]	
MV-NIS (Measles Virus Strain)		N/A—OV monotherapy; evaluated for potential future ICI combination		Recurrent Pediatric CNS Tumors (AT/RT, Medulloblastoma)		Phase I		Single-arm, dose-escalation, first-in-pediatric-population			None		Safety and tolerability		Early feasibility/safety data in a rare pediatric population; ICI combination not yet tested clinically	Exploratory/preclinical rationale only		[36]	

Abbreviations: anti-CTLA-4—anti-cytotoxic T-lymphocyte-associated protein 4; anti-PD-1—anti-programmed cell death protein 1; AT/RT—atypical teratoid/rhabdoid tumor; CI—confidence interval; CNS—central nervous system; CR—complete response; CVA21—coxsackievirus A21; DOR—duration of response; HR—hazard ratio; ICAM-1—intercellular adhesion molecule 1; ICI—immune checkpoint inhibitor; mRANO—modified Response Assessment in Neuro-Oncology; MV-NIS—measles virus encoding the sodium iodide symporter; N—number of patients; N/A—not applicable; OR—odds ratio; ORR—objective response rate; OS—overall survival; OV—oncolytic virus; PFS—progression-free survival; RECIST v1.1—Response Evaluation Criteria in Solid Tumors version 1.1; RP1, vusolimogene oderparepvec; T-VEC—talimogene laherparepvec.

**Table 5:** OV-ICI combinations in selected cancers and candidate biomarkers for response to therapy.

OV + ICI Combination	Cancer Type	Candidate Biomarkers	What Did Biomarkers Signal	Evidence
**T-VEC +** **pembrolizumab**	Advanced melanoma	Post-OV CD8+, T-cell infiltration, PD-L1 upregulation, IFN-γ expression	Immune priming	[31,151,152]
**T-VEC + ipilimumab**	Unresectable melanoma	Inflamed TME, systemic immune activation	Better ORR than ipilimumab alone	[151,152]
**ONCOS-102 + pembrolizumab**	Anti-PD-1-resistant advanced melanoma	Persistent T-cell infiltration, immune-related gene expression, cytotoxic CD8+ cells	Clinical benefit in resistant disease	[116,153]
**DNX-2401 + pembrolizumab**	Recurrent glioblastoma	TME medium subtype, PDCD1 expression, exploratory tumor mutational burden	Clinical benefit and longer survival	[116,117]
**Pelareorep + pembrolizumab**	Advanced pancreatic ductal adenocarcinoma	T-cell infiltration, PD-L1+ cells, reduced Foxp3+ Tregs	On-treatment immune activation	[31,91]
**Pelareorep + pembrolizumab + chemotherapy**	Advanced pancreatic ductal adenocarcinoma	Peripheral T-cell clonality, CCR5 pathway upregulation as negative biomarker	Longer survival or possible resistance	[31,154]

Abbreviations: OV: oncolytic virus; ICI: immune checkpoint inhibitor; PD-L1: programmed death-ligand 1; IFN-γ: interferon gamma; TME: tumor microenvironment; ORR: objective response rate; PDCD1: Programmed Cell Death Protein 1; CCR5: C-C chemokine receptor type 5.

## Data Availability

No new data were created or analyzed in this study. Data sharing is not applicable to this article.

## Abbreviations

The following abbreviations are used in this manuscript:

AT/RT	atypical teratoid/rhabdoid tumor
ATP	adenosine triphosphate
CAF	cancer-associated fibroblast
CCL	C-C motif chemokine ligand
CI	confidence interval
CNS	central nervous system
CR	complete response
CRT	calreticulin
CTLA-4	cytotoxic T-lymphocyte-associated protein 4
CVA21	coxsackievirus A21
CXCL	C-X-C motif chemokine ligand
CXCR	C-X-C motif chemokine receptor
DAMPs	damage-associated molecular patterns
DC	dendritic cell
DNAM-1	DNAX accessory molecule 1
ECM	extracellular matrix
EGFR	epidermal growth factor receptor
FAP	fibroblast activation protein alpha
FDA	U.S. Food and Drug Administration
FOXP3	forkhead box P3
GM-CSF	granulocyte–macrophage colony-stimulating factor
GSDME	gasdermin E
GZMB	granzyme B
HER2	human epidermal growth factor receptor 2
HMGB1	high-mobility group box 1
HR	hazard ratio
HSV	herpes simplex virus
HSV-1	herpes simplex virus type 1
ICAM-1	intercellular adhesion molecule 1
ICD	immunogenic cell death
ICI	immune checkpoint inhibitor
IDO	indoleamine 2,3-dioxygenase
IFN	interferon
IFN-α	interferon alpha
IFN-β	interferon beta
IFN-γ	interferon gamma
IL	interleukin
ISG	interferon-stimulated gene
JAK	Janus kinase
MAPK	mitogen-activated protein kinase
MDSC	myeloid-derived suppressor cell
MHC	major histocompatibility complex
MV	measles virus
MV-NIS	measles virus expressing the sodium iodide symporter
MXRA8	matrix remodeling-associated protein 8
MYD88/MyD88	myeloid differentiation primary response 88
NAb	neutralizing antibody
NF-κB	nuclear factor kappa-light-chain-enhancer of activated B cells
NIS	sodium iodide symporter
NK	natural killer
NLRP3	NOD-, LRR- and pyrin domain-containing protein 3
OAS	2′-5′-oligoadenylate synthetase
ORR	objective response rate
OS	overall survival
OV	oncolytic virus
OVM	oncolytic virus M1
OVT	oncolytic virotherapy
PAMPs	pathogen-associated molecular patterns
PCR	polymerase chain reaction
PD-1	programmed cell death protein 1
PD-L1	programmed death-ligand 1
PFS	progression-free survival
PI3K	phosphoinositide 3-kinase
PKR	protein kinase R
PMN-MDSC	polymorphonuclear myeloid-derived suppressor cell
PRISMA	Preferred Reporting Items for Systematic Reviews and Meta-Analyses
PRKRA	protein activator of interferon-induced protein kinase EIF2AK2
RP1	vusolimogene oderparepvec
SCD	super cytosine deaminase
SCLC	small-cell lung carcinoma
STAT	signal transducer and activator of transcription
STING	stimulator of interferon genes
SVV	Seneca Valley virus
TAM	tumor-associated macrophage
TEM8/ANTXR1	tumor endothelial marker 8/anthrax toxin receptor 1
TGF-β	transforming growth factor beta
TLR	Toll-like receptor
TME	tumor microenvironment
TNF	tumor necrosis factor
TPRA1	transmembrane protein adipocyte-associated 1
TRAIL	tumor necrosis factor-related apoptosis-inducing ligand
Treg	regulatory T cell
T-VEC	talimogene laherparepvec
VSV	vesicular stomatitis virus
ZAP	zinc-finger antiviral protein
